# Crystal orientation-dependent etching and trapping in thermally-oxidised Cu_2_O photocathodes for water splitting†

**DOI:** 10.1039/d1ee03696c

**Published:** 2022-03-23

**Authors:** Wenzhe Niu, Thomas Moehl, Pardis Adams, Xi Zhang, Robin Lefèvre, Aluizio M. Cruz, Peng Zeng, Karsten Kunze, Wooseok Yang, S. David Tilley

**Affiliations:** Department of Chemistry, University of Zurich Winterthurerstrasse 190 8057 Zurich Switzerland david.tilley@chem.uzh.ch; PERA Complexity (Processes for Evolutionary Complexity Research & Applications) B. V. Hoogoorddreef 15 1101 BA Amsterdam The Netherlands; Scientific Center for Optical and Electron Microscopy (ScopeM) ETH Zürich Otto-Stern-Weg 3 8093 Zurich Switzerland; School of Chemical Engineering, Sungkyunkwan University (SKKU) Suwon Korea

## Abstract

Ammonia solution etching was carried out on thermally-oxidised cuprous oxide (TO-Cu_2_O) in photocathode devices for water splitting. The etched devices showed increased photoelectrochemical (PEC) performance compared to the unetched ones as well as improved reproducibility. −8.6 mA cm^−2^ and −7 mA cm^−2^ photocurrent density were achieved at 0 V and 0.5 V versus the reversible hydrogen electrode (V_RHE_), respectively, in the champion sample with an onset potential of 0.92 V_RHE_ and a fill factor of 44%. An applied bias photon-to-current efficiency of 3.6% at 0.56 V_RHE_ was obtained, which represents a new record for Cu_2_O-based photocathode systems. Capacitance-based profiling studies showed a strong pinning effect from interfacial traps in the as-grown device, and these traps were removed by ammonia solution etching. Moreover, the etching procedure gave rise to a diverse morphology of Cu_2_O crystals based on the different crystallographic orientations. The distribution of crystallographic orientations and the relationship between the crystal orientation and the morphology after etching were examined by electron backscatter diffraction (EBSD) and scanning electron microscopy (SEM). The high-index crystal group showed a statistically higher PEC performance than the low-index group. X-ray photoelectron spectroscopy (XPS) and transmission electron microscopy (TEM) revealed metallic copper at the Cu_2_O/Ga_2_O_3_ interface, which we attribute as the dominant trap that limits the PEC performance. It is concluded that the metallic copper originates from the reduction of the CuO impurity layer on the as-grown Cu_2_O sample during the ALD process, while the reduction from Cu_2_O to Cu is not favourable.

## Abstract 2

Crystal orientation-dependent etching, charge-carrier trapping behaviour, and performance of single-crystal photocathodes are enabled by ammonia solution etching of thermally-oxidised Cu_2_O.

## Graphical abstract

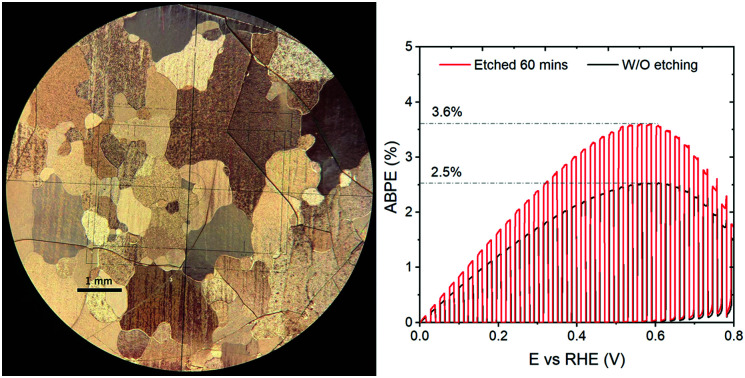

**Broader context**

The global goal of carbon neutrality has increased the demands for sustainable and environmentally-friendly energy sources and energy carriers. Producing hydrogen through water splitting driven by solar illumination with the photoelectrochemical (PEC) method is an attractive strategy. To achieve cost-effective hydrogen production from this method, the efficiency and the stability of the light absorber materials must be high, and to reach a large scale, the PEC devices should be prepared from abundant elements and fabricated in a simple and low-cost manner. Cuprous oxide (Cu_2_O) is a promising p-type semiconductor material that could meet the above requirements if the efficiency could be improved. In this work, ammonia solution etching was adopted to eliminate the surface traps on thermally-oxidised Cu_2_O, thereby improving the hydrogen production efficiency. The etching procedure revealed a crystal orientation dependence on the performance of the photocathode, related to interface states at the Cu_2_O/Ga_2_O_3_ heterojunction, which comprises the photovoltage-generating junction. This discovery points the way to further improvements with Cu_2_O towards practical water splitting cells.

## 1 Introduction

In the past few years, many countries worldwide have declared their road map for reducing carbon dioxide (CO_2_) emissions to achieve net-zero emission by 2050.^1^ Most CO_2_ emissions are from energy consumption by traditional energy sources such as oil and coal.^2^ Finding alternatives to fossil fuels is crucial to achieving these goals. Hydrogen (H_2_), a potential carrier of clean energy, has attracted much attention from researchers. Unfortunately, the large majority of hydrogen used now is still produced from fossil fuels.^3^ Producing H_2_ by photoelectrochemical (PEC) water splitting is a very promising strategy with the help of sunlight.^4–6^

To achieve efficient PEC water splitting, a suitable band gap and good electronic properties are essential for light absorption and photo-generated electron transport.^7,8^ Cu_2_O, as an intrinsic p-type semiconductor with a band gap of 2.1 eV, has been widely studied in the past decades for photovoltaic devices.^9–11^ In addition, the conduction band edge of −0.7 eV versus RHE makes it a promising candidate material as a photocathode for water splitting.^12^ Paracchino *et al.* reported a Cu_2_O-based photocathode with a photocurrent density of −7.6 mA cm^−2^ at 0 V_RHE_ and an onset potential of 0.35 V_RHE_.^13^ In the following years, the PEC performance has been improved to −10 mA cm^−2^ photocurrent density at 0 V_RHE_ and over 0.8 V_RHE_ onset potential, benefitting from a nanostructuring approach combined with a wide band gap semiconductor material as a buffer layer, albeit with a poor fill factor.^14–16^ Correspondingly, the applied bias photon-to-current efficiency (ABPE) is modest and normally has a relatively low peak position *versus* RHE, which hinders its application in unbiased water splitting systems.

The most studied methods to fabricate Cu_2_O include electrodeposition,^17,18^ thermal oxidation,^19,20^ and reactive sputtering.^21,22^ Among them, thermal oxidation is an effective approach to grow a high-quality Cu_2_O sheet with large grain sizes up to millimetres. In our previous research, we prepared a thermally-oxidised cuprous oxide (TO-Cu_2_O) photocathode and studied its unique carrier transport behaviour.^23^ However, the photocurrent and fill factor left room for improvement. Traps on the surface of the TO-Cu_2_O (which become the interfacial traps in the multilayer photocathode device) could be one of the factors that hurt its photocurrent and fill factor.^24^ Chemical etching is a facile and effective way to remove surface traps.^25,26^ Minami *et al.* applied a three-step etching procedure to remove the CuO layer when preparing a TO-Cu_2_O based solar cell.^27^ Ammonia solution was used in etching electrodeposited Cu_2_O film in solar cell devices, which improved both the photovoltage and photocurrent.^26^ However, the mechanism of the improvement has been rarely discussed.

In this paper, the TO-Cu_2_O sheets were etched by ammonia solution to remove any contamination on the as-grown Cu_2_O before the atomic layer deposition (ALD) process (Fig. S1, ESI†). The etching procedure significantly boosted the photocurrent density and fill factor. With capacitance-based techniques including electrochemical impedance spectroscopy (EIS), the effect of the surface-related traps on the carrier transport was analysed. Etching of the sheets revealed a diverse morphology that depends on the crystal orientation of the grains. By classifying the crystal orientation using electron backscatter diffraction (EBSD) mapping and scanning electron microscopy (SEM), we further compared the PEC performance of TO-Cu_2_O photocathodes based on high-index and low-index crystals. Depth-profiling X-ray photoelectron spectroscopy (XPS) and transmission electron microscopy (TEM) revealed the evolution of surface traps during the etching and ALD processes. With a higher ABPE at more positive applied voltage, this work further promotes the application of Cu_2_O-based photocathodes in an unbiased water splitting system and elucidates the possible ways to further improve its performance.

## 2 Results and discussion

### 2.1 PEC performance of the etched TO-Cu_2_O photocathode

**Fig. 1:**
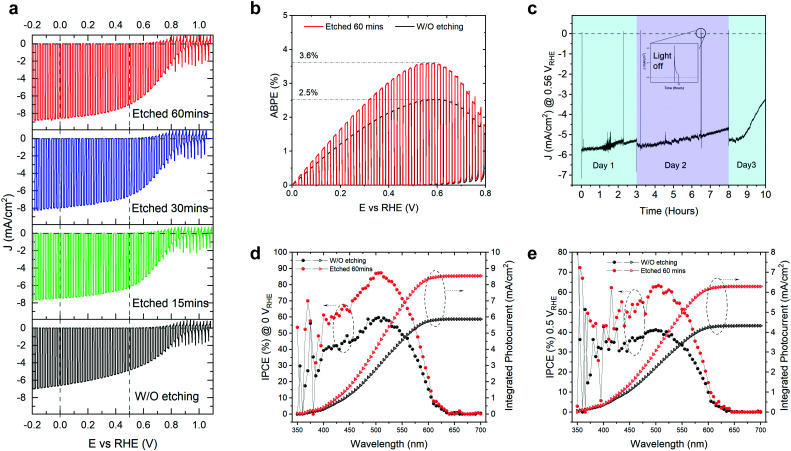
(a) The *J–V* curves of champion Cu_2_O/Ga_2_O_3_/TiO_2_/RuO_*x*_ photocathodes obtained with different ammonia solution etching times under simulated AM 1.5 G irradiation (100 mW cm^−2^) in pH 7 electrolyte. (b) The comparison of ABPE of the unetched and etched (60 min) champion photocathodes. (c) The stability test of an etched sample (60 min) at 0.56 V_RHE_ under simulated AM 1.5 G irradiation (100 mW cm^−2^) in pH 7 electrolyte. The IPCE and integrated photocurrent density of the unetched and etched (60 min) at (d) 0 V_RHE_ and (e) 0.5 V_RHE_.

The as-grown TO-Cu_2_O sheets were immersed in concentrated ammonia solution for different times at room temperature (details can be found in the experimental section and Fig. S1 of the ESI†). The improvement of the PEC performance by the ammonia solution etching is noticeable, as shown in Fig. 1a. The champion photocathode device with 60 minutes of etching time has a photocurrent density of −8.6 mA cm^−2^ and −7 mA cm^−2^ at 0 V_RHE_ and 0.5 V_RHE_, respectively, while without etching the values are −6.6 mA cm^−2^ and −4.8 mA cm^−2^. Statistical data for devices fabricated with 0 (unetched), 15, 30, and 60 minutes of etching are shown in Fig. S2 (ESI†). The photocurrent density at both 0 V_RHE_ and 0.5 V_RHE_ increases at longer etching times, plateauing at 60 minutes. The onset potential does not show a clear trend. Since the onset potential is quite sensitive to the thickness of the Ga_2_O_3_ layer and the thickness of the Ga_2_O_3_ shows small variations, it is presumed that the etching has no apparent effect on the onset potential. Compared with the unetched samples, the etched samples have a higher fill factor.

With 60 minutes of etching, the ABPE increased from 2.5% to 3.6% (Fig. 1b). The ABPE peak at 0.56 V_RHE_ is significant when making tandem devices for unbiased water splitting and represents a new record (Table S1, ESI†). The stability was measured under one sun at 0.5 V_RHE_ (Fig. 1c). The photocurrent density under continuous light decreased slightly on day 1 and day 2, but it recovered after changing the electrolyte or removing the bubbles from the surface of the photocathode. The decrease in the first two days could be due to the attached bubbles on the surface of the device (see Fig. S3, ESI†). The rapid decrease of photocurrent after nine hours of test is due to the real failure of the device. The corrosion of the photocathode mostly happens at the grain boundary of the TO-Cu_2_O, which is visible to the naked eye due to the large grain sizes in the device. Thus, the stability does not show obvious improvement compared with the unetched device in our previous publication.^23^ In Fig. 1d and e, we compared the incident photon-to-current efficiency (IPCE) of the unetched and etched photocathodes. After etching, the IPCE is increased at all wavelength regions from 625 nm to 350 nm. The absolute gain is almost the same. However, the relative increase in the blue region is higher than that of the green region. At 0.5 V_RHE_, the IPCE at 525 nm can exceed 60%, which is crucial to preparing a tandem device for unbiased overall water splitting.

### 2.2 Interfacial traps study by capacitance techniques

According to the cross-sectional SEM image of the etched TO-Cu_2_O (Fig. S4, ESI†), the thickness change during etching is negligible compared to the thickness of the Cu_2_O sheet. Thus, the PEC gain does not benefit from the decreased thickness of the Cu_2_O sheet, which is discussed in our previous publication.^23^ Yet the etching change on the surface of the TO-Cu_2_O sheet plays a vital role in the improvement. To evaluate the change in surface trap density (interfacial traps in multilayer fabricated photocathode device), capacitance–voltage profiling (*C*–*V* profiling) and drive-level-capacitance profiling (DLCP) measurements were performed in the dark on two diode devices (unetched and etched) with the structure Au/TO-Cu_2_O/Ga_2_O_3_/TiO_2_/Ag (Fig. S5, ESI†).

The standard capacitance profiling data can provide the charge carrier density at the Cu_2_O/Ga_2_O_3_ interface. However, it cannot distinguish between bulk and interface defects, since both types of defects respond to this type of measurement. In contrast, DLCP is normally assumed to be insensitive to interfacial traps. By comparing these two measurements, we could estimate the traps from the interface that the ammonia solution etching could remove. This technique has been used to analyse the defects in Si,^28^ CuIn_1−*x*_Ga_*x*_Se_2_,^29^ Cu_2_ZnSnSe_4_,^30^ and perovskite^31^ solar cells.

**Fig. 2:**
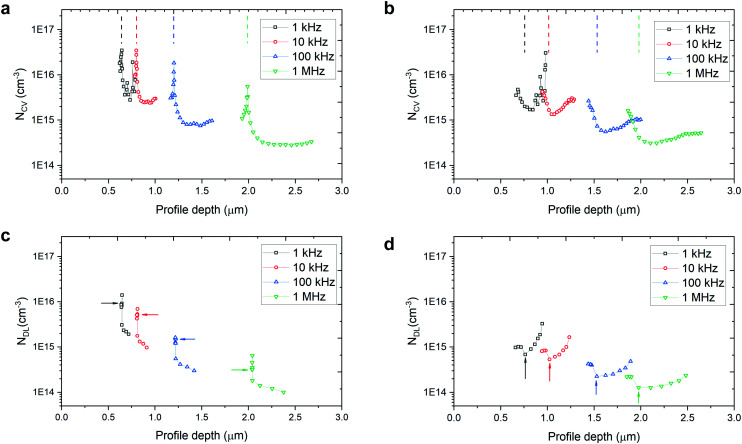
(a), (b) *C*–*V* and (c), (d) DLCP profiles for different frequencies at room temperature of (a), (c) unetched and (b), (d) etched samples. The dashed line in (a), (b) and the arrow in (c), (d) represent the profile position at zero bias at each frequency.

Fig. 2a and b shows the *C*–*V* charge density-profile depth (*N*_CV_ − 〈*x*〉) plots and Fig. 2c and d gives the DLCP charge density-profile depth (*N*_DL_ − 〈*x*〉) plots, extracted from the raw data of *C*–*V* measurement (Fig. S6, ESI†) and DLCP measurement (Fig. S7, ESI†). The details for extracting the data in Fig. 2 can be found in the ESI.† The charge density at *V*_DC_ = 0 V was chosen to estimate the free carrier and trap density.

In the capacitance–voltage plots in Fig. S6 (ESI†), the capacitance of the unetched sample is obviously pinned from −0.1 V to 0.2 V while that of the etched sample changes smoothly as the applied voltage increases (from reverse to forward bias) over the same region due to the narrowing width of the space charge region on the Cu_2_O side. Correspondingly, the sharp peak at around 0 *V*_DC_ bias for each frequency is a clear difference between the unetched and etched devices. Since the etching very likely only changes the surface properties of the TO-Cu_2_O, the pinning effect is related to the traps at the Cu_2_O/Ga_2_O_3_ interface. The peak of the charge density is stable at around 3.5 × 10^16^ cm^−3^ for the AC frequency 10 kHz and below. When the diodes were interrogated with an AC bias at 1 MHz, both the etched and unetched diodes showed a charge density of around 3 × 10^14^ cm^−3^, which is slightly higher than the free carrier density of the TO-Cu_2_O measured by Hall measurement due to the presence of the interfacial traps.^23^

In theory, DLCP is not sensitive to the interfacial traps. However, in our case, the interfacial traps still significantly affect the measured charge density, as depicted in Fig. 2c. What is noticeable here is that the pinning effect of the capacitance at around 0 *V*_DC_ bias can also be observed in the DLCP (Fig. S7, ESI†), while in the etched sample there is almost no peak shown in the near-zero bias region (Fig. 2d). Without the interference of interfacial traps, the charge density is 1.5 × 10^14^ cm^−3^, derived from *N*_DL_ at 1 MHz (*N*_DL,1MHz_), which is regarded as the free carrier density in bulk TO-Cu_2_O and matches well with the value in the literature. Table 1 summaries the *N*_DL_ and *N*_CV_ at different frequencies at *V*_DC_ = 0 V. By subtracting *N*_DL,1MHz_ from *N*_DL,1kHz_ of the etched sample, the trap density in the bulk TO-Cu_2_O is estimated to be 4.5 × 10^14^ cm^−3^. The interfacial trap density can be calculated by subtracting N_DL,1kHz_ of the etched sample from *N*_CV,1kHz_ of the unetched or etched samples. The values for the unetched and etched samples are 3.4 × 10^16^ cm^−3^ and 9.0 × 10^14^ cm^−3^, respectively. After etching, the interfacial trap density is markedly reduced.

**Table 1:** Summary of *N*_DL_ and *N*_CV_ at different AC frequencies at *V*_DC_ = 0 V

Sample	*N*_DL,1MHz_ (cm^−3^)	*N*_DL,1kHz_ (cm^−3^)	*N*_CV,1kHz_ (cm^−3^)	Free carrier density (cm^−3^)	Trap density in bulk (cm^−3^)	Trap density at interface (cm^−3^)
Unetched	3.0 × 10^14^	9.0 × 10^14^	3.5 × 10^16^	1.5 × 10^14^	4.5 × 10^14^	3.4 × 10^16^
Etched	1.5 × 10^14^	6.0 × 10^14^	1.5 × 10^15^			9.0 × 10^14^

Additionally, EIS was applied to study the charge carrier behaviour in pH 7 solution under 10% sunlight intensity. The equivalent circuit model used to fit the data after the photocurrent onset consists of 3 RC elements in series (plus a series resistance) for the etched sample. In the unetched sample, the equivalent circuit contained 4 RC elements plus the series resistance (Fig. S8a, ESI†). CPE elements were used to model the capacitances with the ideality factor not going below 0.8. The Nyquist and the Bode plots (Fig. S8b, c and S9a, ESI†) obtained from the EIS of samples at 0 V_RHE_ and −200 mV_RHE_ show 3 and 4 elements, respectively. Fig. S9b (ESI†) shows the extracted resistances and capacitances from the fitting procedures. The observed elements in the equivalent circuit are not assigned to a specific (photo)physical process. However, we observe that even though the devices are fabricated similarly with only one additional etching step of the Cu_2_O surface, the main difference in the fitting results is the additional RC element in the unetched sample (Fig. S9b, c (ESI†), R_MF2 and Tau_MF2). As the *JV* curves under 10% sunlight for the etched and unetched samples differ mainly in the FF, the reduction of the FF in the unetched sample is probably based on this additional resistive element, representing a series resistance for the photogenerated charges.

### 2.3 Single-crystal TO-Cu_2_O photocathodes

**Fig. 3:**
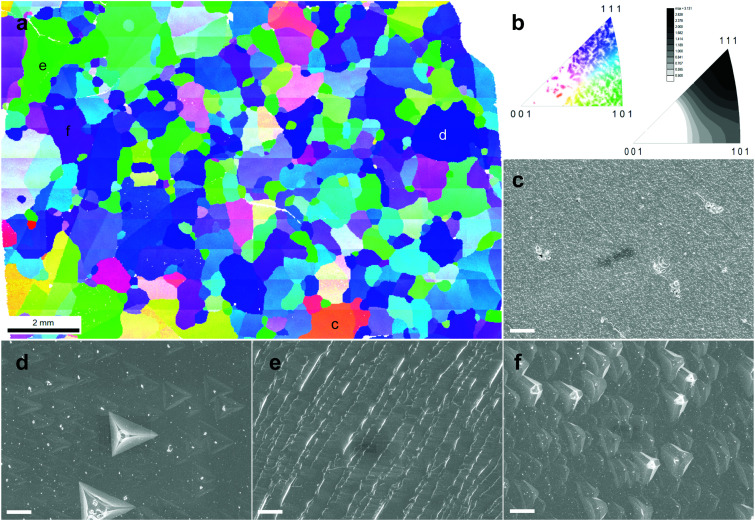
(a) The orientation map shown with the inverse pole figure (IPF) colour key with respect to the surface normal direction of an etched TO-Cu_2_O. (b) The coloured figure is the legend of orientation and the grey figure with the scale bar shows the orientation density in the inverse pole figure with respect to the surface normal direction. (c–f) SEM images of corresponding sites in (a) with typical crystal orientations. The scale bar in (c–f) is 10 micrometres.

The morphology of the etched sample drew our attention. Generally, the surface of the as-grown TO-Cu_2_O is smooth with a uniform colour, whilst that of the etched sample shows a different morphology at different crystal facets (Fig. S10, ESI†). To investigate the relationship between the morphology after etching and the crystallographic orientation, we performed EBSD measurements on an etched TO-Cu_2_O sheet. Fig. 3a and b shows the orientation map and the grain orientation distribution. The polycrystalline sheet is strongly textured, dominated by grains with (111) or (101) planes parallel to the surface of the Cu_2_O sheet, while almost no crystal with [001] orientation was found. Single-crystal XRD analysis of a portion of one of these domains (sample size 0.52 × 0.39 × 0.075 mm^3^) confirms that the uni-coloured domains of up to several millimetres in Fig. 3 are indeed single-crystalline, as shown in Fig. S11 (ESI†).

By measuring the SEM image of each site, we can determine the relationship between morphology and orientation. Typically, the deep blue grains with [111] orientation have a flat surface with some regular pyramidal pits (Fig. S12, ESI†). The shape of the pit matches well with a negative imprint of the corner of the cubic crystal structure of Cu_2_O. At the green site, regular ridge-shaped humps are related to the edge of the cubic crystal (Fig. S13, ESI†). Another typical morphology is found at the purple site with [hh1] orientation (typically [112] or [223]), which is also flat but with some shallow isosceles triangular pyramid-shaped hollows (Fig. S14, ESI†). The grains with other colours, far from the corner or edge of the unit triangle in Fig. 3b, have a more diverse morphology. We selected some sites to show the morphology in Fig. S15 (ESI†). With the crystal orientation and SEM morphology, these crystals could be roughly divided into two groups: the low index group (LIG), including the blue, green and purple sites, with (111), (101), and (hh1) planes parallel to the surface of the Cu_2_O sheet, respectively; and the high index group (HIG), including all crystals with other colours, which has a high index plane parallel to the surface.

**Fig. 4:**
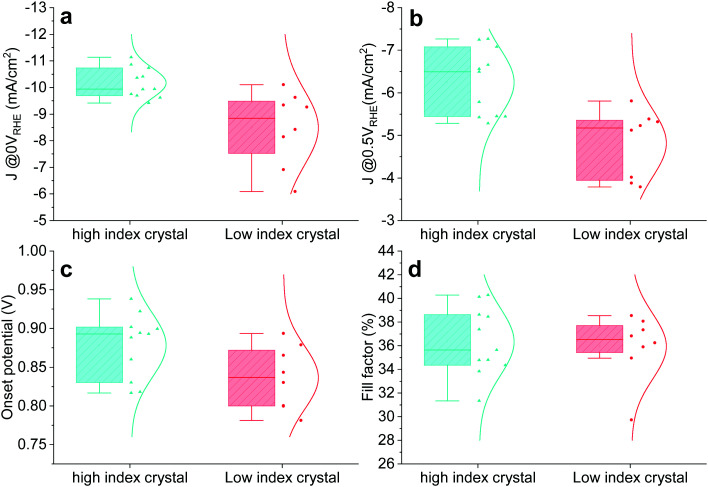
Statistical PEC performance of high index (HIG) and low index (LIG) single crystal photocathodes. Photocurrent at (a) 0 V_RHE_ and (b) 0.5 V_RHE_. (c) Onset potential. (d) Fill factor.

The crystal size of Cu_2_O grown by thermal oxidation is up to millimetres. Thus, it is possible to compare the photocathode device PEC performance in relation to the two groups (HIG and LIG). Samples with a single crystal TO-Cu_2_O sheet after 60 minutes of etching were prepared, as shown in Fig. S16 (ESI†). The reflected and the transmitted light through the edge of the surrounding epoxy cannot be ignored for such a small area photocathode, so we only compare the relative PEC performance between these two groups. To avoid any possibility of preferential photodeposition of catalyst on the different grains, 1 nm Pt was sputtered as the catalyst instead of the RuO_*x*_ in the single-crystal devices. The statistical data of the PEC performance is shown in Fig. 4. The photocurrent density is higher in the high index crystal group than the low index crystal group at 0 V_RHE_ and 0.5 V_RHE_. The IPCE data also shows that the high index device has a much higher quantum efficiency from 400 nm to 550 nm as compared to the low index device at 0 V_RHE_ and 0.5 V_RHE_ (Fig. S17, ESI†). The HIG samples have a higher onset potential than the LIG, with a slightly higher fill factor, due to the diverse microstructures in the high index group.

EIS was performed with two representative single-crystal photocathode devices (HIG and LIG). Three elements could be extracted from the Nyquist plots of both samples (Fig. S18, ESI†), meaning that both samples behave similarly to the large etched sample with both high-index and low-index crystals. The increasing high-frequency resistances (R_HF, red lines) further support that both samples are etched (note that the R_HF of the unetched sample is almost constant over the range of potentials used, Fig. S9, ESI†). Despite their similarities, the R_HF of the low index sample is relatively low, while the high index device is almost identical to the etched sample presented in Fig. S9 (ESI†). The discrepancy between the high and low index samples indicates that the etching process is more effective for the high index sample, implying selective etching depending on the crystal orientation. The selective etching is further supported by the lifetime of the high-frequency arc (Tau_HF, Fig. S18b, ESI†) as well as the performance differences in Fig. 4. The *C*–*V* profiling experiments were also conducted on high-index and low-index single-crystal devices, as shown in Fig. S19 (ESI†). The derived charge density showed that the interfacial traps in both low-index and high-index devices were reduced to some extent. The high-index device had a lower interfacial trap density compared to the low-index device, which is consistent with the observed PEC performance and the EIS analysis.

### 2.4 The origin of the interfacial traps

**Fig. 5:**
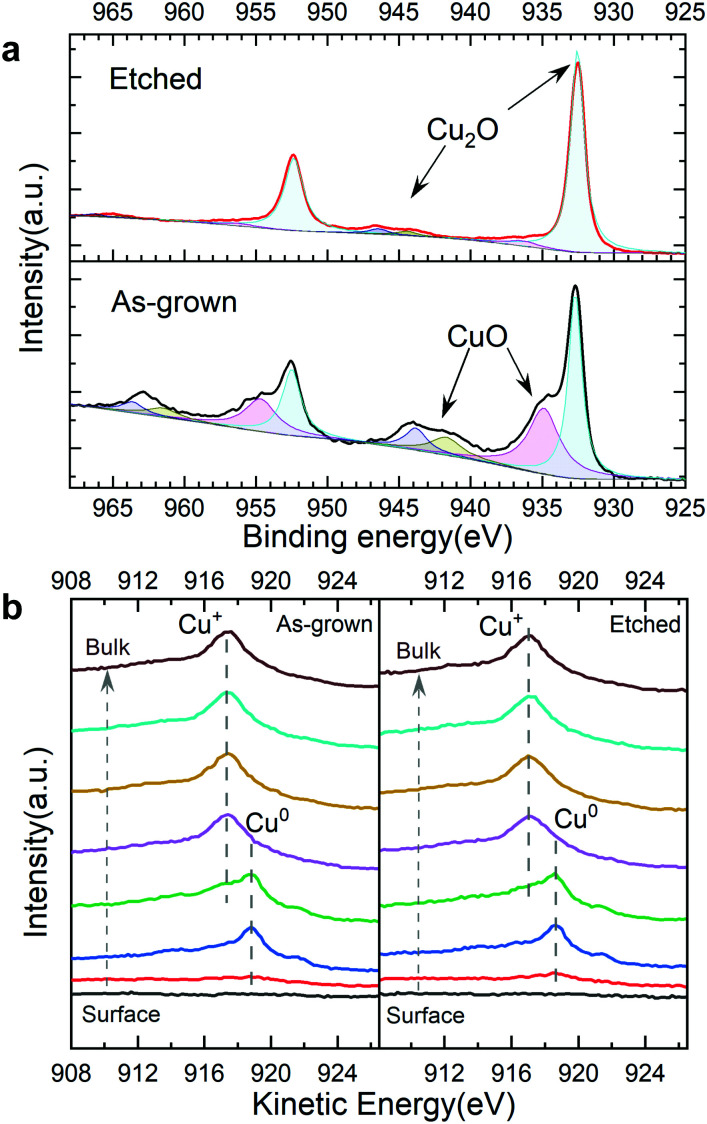
(a) The surface XPS Cu 2p region of as-grown and etched TO-Cu_2_O. (b) Depth profile XPS of the Cu LMM Auger peak of as-grown and etched TO-Cu_2_O with a 5 nm ALD Ga_2_O_3_ layer.

To further reveal the effect of the ammonia solution etching, depth profile XPS was performed on the bare TO-Cu_2_O samples with and without etching (Fig. S20, ESI†). From the Cu 2p peak at 932.6 eV and the Cu LMM Auger peak at 916.9 eV, we can confirm that Cu^+^ is the only existing state in the bulk of TO-Cu_2_O. However, two apparent features of CuO – the Cu 2p shoulder peak at around 934.9 eV and the broad satellite peak from 940 eV to 945 eV (Fig. 5a) – are found at the surface of the as-grown TO-Cu_2_O. After etching, only the peak at around 932.6 eV was detected, which is from the Cu_2_O. In addition, the weak satellite peak from 943 eV to 947 eV is another typical feature of Cu_2_O. In the depth profile XPS study of the bare TO-Cu_2_O, the peak that belongs to CuO disappeared after only brief sputtering (1 minute), which means the CuO layer is relatively thin.

Depth profile XPS on two TO-Cu_2_O samples (with and without etching) with a 5 nm ALD Ga_2_O_3_ layer were conducted to reveal the copper oxidation states at the interface of Cu_2_O and Ga_2_O_3_ in the photocathode device (Fig. S21, ESI†). Compared with the etched sample, the Cu 2p showed a small shoulder peak at 934 eV, possibly from CuO. However, from Cu LMM Auger peaks, no clear difference was observed. At the interface of Cu_2_O/Ga_2_O_3_, a clear metallic copper peak at 918.7 eV was observed in both samples. The small shoulder peak at 921.4 eV is another typical feather of the Cu^0^ (Fig. 5b). Two Cu_2_O samples with a 5 nm Au layer on the surface were prepared to investigate the origin of the metallic copper. The Cu LMM Auger data in the depth profile XPS showed only a small peak for Cu^2+^ (918.0 eV), but no metallic copper (Cu^0^) at the interface of the Cu_2_O and Au (Fig. S22, ESI†). Thereby, it can be concluded that metallic copper is formed during the ALD process of the Ga_2_O_3_ deposition.

Cross-sectional TEM images were obtained to investigate the microstructure at the Cu_2_O/Ga_2_O_3_ interface. The cross-sectional TEM lamellae were prepared by focused ion beam (FIB). To get two different crystal sites in one lamella, the lamellae were cut and lifted out by FIB along the red line crossing the grain boundary of two crystals (Fig. S23, ESI†). In the etched sample, one side is the high index area, while the other side belongs to the low index area, which could be distinguished by the surface morphology. In the unetched sample, it was not possible to determine the crystal orientation by morphology.

**Fig. 6:**
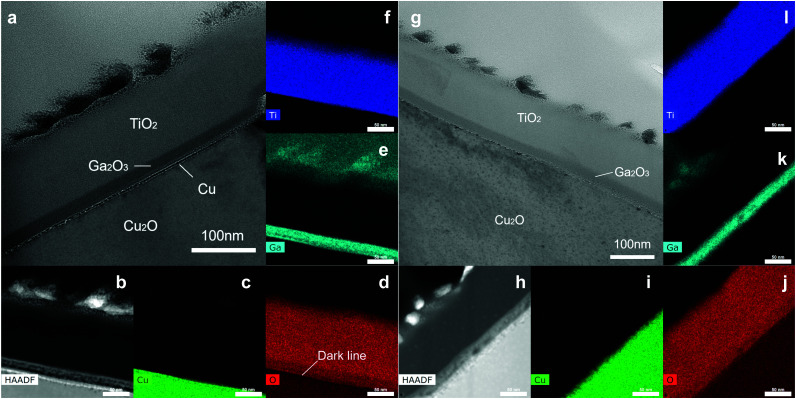
The cross-sectional STEM bright-field image (a), STEM high-angle annular dark-field image (b) and elemental maps (c–f) of Cu, O, Ga, Ti, respectively, of the unetched photocathode device. The cross-sectional STEM bright-field image (g), STEM high-angle annular dark-field image (h) and elemental maps (i–l) of Cu, O, Ga, Ti, respectively, of a high index area in the etched photocathode device. The scale bar in b–e and h–j is 50 nm.

As shown in Fig. 6a, the thickness of the TiO_2_ and the Ga_2_O_3_ layers match well with that observed on the reference silicon wafer of the ALD process. In the STEM bright-field image, the RuO_*x*_ dots are the darkest, while the TiO_2_ layer is brightest due to mass contrast. Notably, a clear dark line (or dots) at the Cu_2_O/Ga_2_O_3_ interface was observed, indicating the existence of a thin layer. This observation, along with the depth profile XPS data, suggests that the dark line is most likely thin metallic copper. The EDS maps confirmed this as shown in Fig. 6b–f. An obvious dark line, corresponding to an oxygen-poor area, could be observed in the O map (Fig. 6d) at the interface of the Cu_2_O and Ga_2_O_3_. This metallic Cu layer could also be confirmed in the high-angle annular dark-field STEM image, which showed a bright contrast in the HAADF STEM image (Fig. 6b). In the STEM bright-field image from the high index area of the etched sample, even though there is still a clear similar dark area, no obvious bright contrast is observed in the HAADF STEM image (Fig. 6h) and the O mapping image does not show an oxygen-poor area (Fig. 6j), indicating no metallic Cu layer here. However, in the low index area, this oxygen-poor area has again appeared. In Fig. S24 (ESI†), we show the HAADF and EDS mapping in these four sites. The metallic copper layer in the etched device is reduced to some extent.

In Table S2 (ESI†), we summarize the XPS and TEM data in the etched and unetched samples. In the unetched sample, a thin CuO layer is formed during the oxidation process of copper foil. However, the CuO is not the origin of the interfacial traps in the device, but metallic copper, which arises from the reduction of the CuO layer by the ALD precursor. In the etched sample, in general, less metallic copper was detected compared with the unetched sample. The high index area is more effectively etched than the low index area, consistent with the PEC performance. Many literature reports have indicated that reducing CuO is more favourable than reducing Cu_2_O. Moreover, the CuO is preferentially reduced directly to metallic Cu without forming intermediates or suboxides.^32–34^ This offers an explanation for why the as-grown sample with the CuO layer has more metallic copper at the Cu_2_O/Ga_2_O_3_ interface. After etching by ammonia solution, the formation of metallic copper from the Cu_2_O is less feasible, and also less CuO is present for the copper formation process.

## 3 Conclusions

Surface etching of Cu_2_O by ammonia solution has proven to be an effective method to boost the PEC performance of TO-Cu_2_O based photocathodes. The champion device achieved a photocurrent density of −8.6 mA cm^−2^ and −7 mA cm^−2^ at 0 V_RHE_ and 0.5 V_RHE_, respectively, and an onset potential of 0.92 V_RHE_, while yielding an ABPE of 3.6%, a new record for Cu_2_O-based photocathode systems. Capacitance–voltage measurements as well as EIS confirm that the interfacial traps at the Cu_2_O/Ga_2_O_3_ interface could be removed by etching, thereby dramatically increasing the carrier lifetime in the etched sample. The revealed morphology by etching is highly correlated to the Cu_2_O grain orientation. The high index group has a much better PEC performance than the low index group, as less metallic copper is formed at the Cu_2_O/Ga_2_O_3_ interface. Metallic copper is the main interfacial trap that reduces the PEC performance in the unetched sample, produced as a result of the reduction of the surface CuO layer during the ALD process. In contrast, the etched sample without a CuO layer is more resistant to this process. This investigation shows how with further engineering of the Cu_2_O surface PEC performance can be improved.

## Author contributions

W. N. and S. D. T. conceived the project. W. N. performed the fabrication of the photocathodes, the PEC performance test, the IPCE test and the capacitance tests. X. Z. assisted parts of the measurements. T. M. and W. Y. conducted and evaluated the EIS experiments. P. A. and W. N. conducted and evaluated the XPS experiments. R. L. conducted and evaluated the SXRD measurements. P. Z. prepared the TEM lamellae by FIB and conducted and evaluated the TEM experiments. K. K. conducted and evaluated the EBSD measurements. A. M. C. supervised the work. W. N. and S. D. T. wrote the manuscript. All the authors contributed to discussions of the results and revisions of the manuscript.

## Conflicts of interest

W. N. received sponsored research funding from PERA Complexity for the work in this article. The methodology described above is part of a system with the potential for commercialization as technology to be led by PERA Complexity within its patent portfolio.

## Supplementary Material

EE-015-D1EE03696C-s001
