# Supplementary material for: Crystal orientation-dependent etching and trapping in thermally-oxidised Cu2O photocathodes for water splitting
Source: Energy Environ Sci. 2022 Mar 23;15(5):2002–10. doi: 10.1039/d1ee03696c (PMC9116084; doi:10.1039/d1ee03696c)
Supplement: EE-015-D1EE03696C-s001 [file EE-015-D1EE03696C-s001.pdf]

Electronic Supplementary Material (ESI) for Energy & Environmental Science.
This journal is © The Royal Society of Chemistry 2022

Supplementary Information

Crystal orientation-dependent etching and trapping in thermally-oxidised Cu_2O photocathodes for water splitting

Wenzhe Niu,[a] Thomas Moehl,[a] Pardis Adams,[a] Xi Zhang,[a] Robin Lefèvre,[a] Aluizio M. Cruz,[b] Peng Zeng,[c] Karsten Kunze,[c] Wooseok Yang,[ad] and S. David Tilley*[a]

a Department of Chemistry, University of Zurich, Winterthurestrasse 190, 8057 Zurich, Switzerland.
b PERA Complexity (Processes for Evolutionary Complexity Research \& Applications) B.V. Hoogoorddreef 15 1101 BA Amsterdam The Netherlands.
c Scientific Center for Optical and Electron Microscopy (ScopeM), ETH Zürich, Otto-Stern-Weg 3, 8093 Zürich, Switzerland.
d School of Chemical Engineering, Sungkyunkwan University (SKKU), Suwon, Korea.
* Email： david.tilley@chem.uzh.ch

Contents

Experimental *2*
Fabrication of TO-Cu_2O photocathode 2
PEC, ABPE, IPCE, and EIS measurement of Cu_2O photocathodes 2
Device fabrication and measurement for CV and DLCP 3
Material Characterization 4
Supplementary figures and tables *5*
Supplementary references *30*

Experimental

Fabrication of TO-Cu_2O photocathode

Growth of Cu_2O sheet by thermal oxidation. The method is the same as our previous paper.[1] The 0.05mm thick Copper foil (99.99%) is bought from Sigma.

Etching of TO-Cu_2O by ammonia solution. The as-grown TO-Cu_2O was etched by concentrated ammonia hydroxide solution (Honeywell, ~25% NH3 basis) for different times. Typically, a 60ml solution was used for eight pieces of TO-Cu_2O samples. After etching, the TO-Cu_2O sheets were rinsed with distilled water several times and then dried carefully under a stream of nitrogen. The etched TO-Cu_2O samples were transferred into the ALD chamber immediately (within a few minutes).

ALD of Ga_2O_3 and TiO_2 layers. The ALD process was operated in a thermal ALD system (PICOSUN, R-200). Bis (μ-dimethylamino)tetrakis(dimethylamino)digallium (STREM, 98%), tetrakis(dimethylamino)titanium (Aldrich, 99.99%), and water were the precursors of Ga, Ti, and O, respectively. The reaction chamber temperatures for Ga_2O_3 and TiO_2 deposition were 160°C and 120°C, respectively. The precursor temperatures of Ga, Ti, and O were kept at 155°C, 85°C, and RT. For Ga_2O_3, the Ga precursor ALD valve was opened for 2.5 s (0.5 s dosing to the reactor, 2.1 s of increased line pressure, i.e "boost" mode), followed by a 7.0 s N_2 purge. Then a 0.1 s pulse of H_2O was used, followed by a 4.0 s N_2 purge. For TiO_2, the Ti precursor ALD valve was opened for 1.6 s (0.5 s dosing to the reactor, 1.2 s of increased line pressure, i.e. "boost" mode), followed by a 6.0 s N_2 purge. Then a 0.1 s pulse of H_2O was used, followed by a 6.0 s N_2 purge. During ALD, the backside and edge of the TO-Cu_2O were protected by Teflon tape.

Packaging of the photocathodes. After the ALD process, a 100nm gold layer was deposited onto the backside of the Cu_2O sheet by a sputter coater (Safematic CCU-010). A copper wire was connected to the gold layer by silver paste. Finally, the Cu_2O was masked and sealed by opaque epoxy, exposing only the working area. The working area was measured with ImageJ software.

Catalyst loading. Ruthenium oxide (RuO_x) or platinum (Pt) were used as the hydrogen evolution reaction (HER) catalyst. RuO_x was photoelectrodeposited under one sun illumination with a constant current density of 28.3 μA cm^{-2} from a 1.3×10^{-3} M potassium perruthenate ($KRuO_4$, Alfa Aesar) solution, as described in the literature.[2] The deposition time was 15 minutes. Pt catalyst was sputtered onto the surface of the fabricated device, monitored by a quartz microbalance to control the thickness.

The overall fabrication procedures are showed in Fig. S1.

PEC, ABPE, IPCE, and EIS measurement of Cu_2O photocathodes

Electrochemical measurements were performed in a three-electrode electrochemical cell in potassium phosphate solution with a pH 7 buffer solution. A Pt wire and an Ag/AgCl electrode (KOSLOW, saturated KCl, +0.197 V vs normal hydrogen electrode (NHE)) were used as counter and reference electrodes. The electrolyte was prepared by dissolving 93.43 g K_2HPO_4 and 63.09 g KH_2PO_4 in 1 L distilled water. All potentials in this paper were converted into reversible hydrogen electrode (RHE) scale using the equation (1).

$$V_{RHE} = V_{Ag/AgCl} + 0.059 \times pH + 0.197 \tag{1}$$

PEC measurement. A potentiostat (Biologic SP-200) was used in the PEC test. The light source was a 150 W Xe-lamp (LOT Oriel) equipped with an AM 1.5 G filter, and the light intensity (100 mW cm^{-2}) was calibrated with a standardized silicon solar cell (PV Measurements, USA). The J-V curves were measured under chopping or continuous light. For statistical data, the photocurrent density at 0 V_{RHE} and 0.5 V_{RHE} is picked in the cyclic voltammetry scan from negative to positive with a scan rate of 30 mV/s under continuous light. The onset potential (V_{onset}) is defined as the intersection of the J-V curve and J = 0 mA cm^{-2}. The fill factor is defined as $\frac{Max(J \times V_{RHE})}{J_{0\,V_{RHE}} \times V_{onset}} \times 100\%$.

ABPE calculation. The ABPE was calculated from the current density-potential curve of the photocathode by the equation (2).[3]

$$ABPE(\%) = \frac{J(mA/cm^2) \times V_{RHE}}{P(mW/cm^2)} \times 100 \quad (2)$$

Where J is the photocurrent density, V is the applied potential, and P is the light intensity (100mW cm^{-2}).

IPCE measurement. IPCE was measured in a home-built system equipped with a halogen light source and a double monochromator. The light intensity was measured with a calibrated silicon photodiode before each measurement. The IPCE was calculated based on equation (3).

$$IPCE(\lambda) = \frac{1240(V \times nm) \times J(mA\ cm^{-2})}{P(mW\ cm^{-2}) \times \lambda(nm)} \times 100\% \quad (3)$$

Where J is the photocurrent density, P is the light intensity at each light wavelength, λ is the wavelength of the monochromatic light. The calculated photocurrents based on the IPCE are slightly lower than the measured photocurrents. One reason for an underestimation is due to the low signal in the UV and blue regions due to low photon flux from the monochromator, as can be seen in the quasi-stochastic response in those regions in Fig 1. Also, bubble adhesion effectively reduces the surface area of the photocathode, resulting in less current flow. The bubble clinging phenomenon is more of a problem under low light intensity conditions, as it takes a long time for the bubble to get large enough to detach.

EIS measurement. The EIS was performed with a potentiostat (Biologic SP-200) with the staircase potential electrochemical impedance spectroscopy (SPEIS) technique. It was scanned from 1 MHz to 0.2 Hz with a stabilization time at the potential applied of 15 s and a modulation voltage of Vrms = 15 mV. Zview is the software used to fitting the EIS data.

Device fabrication and measurement for CV and DLCP

A copper wire was connected to the TiO_2 layer by silver paste instead of loading a catalyst onto the photocathode device. The CV and DLCP were measured with a potentiostat (Biologic SP-200) in a two-electrode configuration. For CV measurement, the DC bias (V_{DV}) was scanned from -1.5 V to 0.5 V, and the amplitude of the AC bias was 5 mV. The frequency of the AC bias was scanned from 1kHz to 1MHz. To calculate the carrier densities from CV (N_{CV}), the following equation (4) was used:

$$N_{CV} = -\frac{2}{q\varepsilon\varepsilon_0 A^2}\left[\frac{d(C^{-2})}{dV_{DC}}\right] \quad (4)$$

Where q is the elementary charge, ε is the dielectric constant of the Cu_2O (taken as 7.6[4]), ε_0 is the permittivity of free space, and A is the area of the diode.

For the DLCP measurement, the DC bias was from -1 V to 0.5 V, while the amplitudes of the AC biases were ranging from 20 mV to 300 mV. An additional offset DC voltage was applied for each AC bias to keep the maximum value of reverse bias ($Max(V_{DC}+V_{AC})$) the same. For example, if a series of data with $Max(V_{DC}+V_{AC})$ = -1 V were measured, the combination of V_{DC} and V_{AC} should be set to (V_{DC} = -1.02 V , V_{AC} = 20 mV), (V_{DC} = -1.04 V , V_{AC} = 40mV), (V_{DC} = -1.06 V , V_{AC} = 60mV), … , (V_{DC} = -1.30 V , V_{AC} = 300mV). To simply, V_{DC} is used in the main text instead of $Max(V_{DC}+V_{AC})$.[5] In DLCP, the derivation of carrier density including deep trap states is based on the non-linear relationship between the change of charges (δQ) and the perturbation AC bias (δV).

$$\frac{\delta Q}{\delta V} = C_0 + C_1 \delta V + C_2 (\delta V)^2 + \dots \tag{5}$$

By fitting with a quadratic function to obtain C_0 and C_1, the carrier density from DLCP (N_{DL}) could be calculated by:

$$N_{DL} \approx \frac{-C_0^3}{2q\varepsilon\varepsilon_0 A^2 C_1} \tag{6}$$

The profiling distance from the junction barrier $\langle x \rangle$ for both CV (using C in equation (4)) and DLCP (using C_0 in equation (5)) is given by the equation (7).

$$\langle x \rangle = \frac{\varepsilon\varepsilon_0 A}{C} \tag{7}$$

Material Characterization

The morphology of the photocathode was observed by a Zeiss Gemini 450 SEM.

The EBSD of the etched TO-Cu_2O was conducted using a SEM (FEI Quanta 200F) equipped with an electron backscatter diffraction camera (Ametek-EDAX Hikari and OIM 8.6). Single-crystal XRD analysis was performed at 160(1) K using Cu Ka1 radiation (l = 1.54184 Å on a XtaLAB Synergy, Dualflex, Pilatus 200K). Pre-experiment, data collection, and manual data reduction were carried out with the program suite CrysAlisPro.

X-ray photoelectron spectroscopy (XPS) was performed using a Physical Electronics (PHI) Quantum 2000 X-ray photoelectron spectrometer featuring monochromatic Al Kα radiation, generated from an electron beam operated at 15 kV and 32.3 W. The energy scale of the instrument was calibrated using Au and Cu reference samples. The analysis was conducted at 1 x 10^{-6} Pa, with an electron take-off angle of 45° and a pass energy of 23.50 eV.

The TEM lamellae of cross-sectional samples of the Cu_2O devices for TEM imaging were prepared by FIB (Helios 5 UX, Thermo Scientific, the Netherland) using AutoTEM 5 (Thermo Scientific, the Netherland). Carbon deposition was used to protect the surface. The chuck milling and lamellae thinning were done at 30 kV with FIB current from 20 nA to 90 pA. Then the lamellae were polished at 5 kV and finished at 2 kV. TEM characterisation was performed by a TEM (Talos F200X, Thermo Fisher Scientific, the Netherland) operating at 200 kV. Both TEM and STEM modes were used. EDS mapping was acquired by using quadrant EDS detectors (Super-X, Thermo Fisher Scientific, the Netherland) in STEM mode.

Supplementary figures and tables

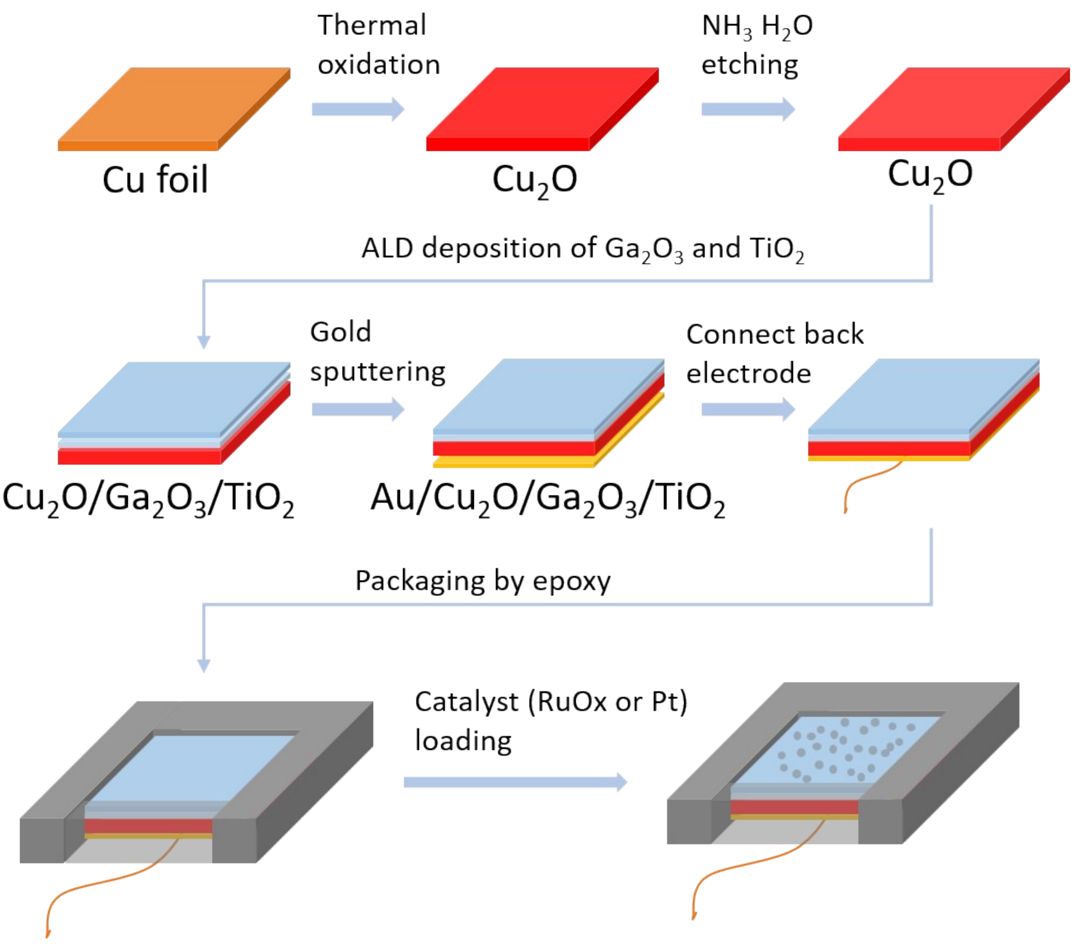

Fig. S 1 The fabrication procedures of TO-Cu_2O photocathode.

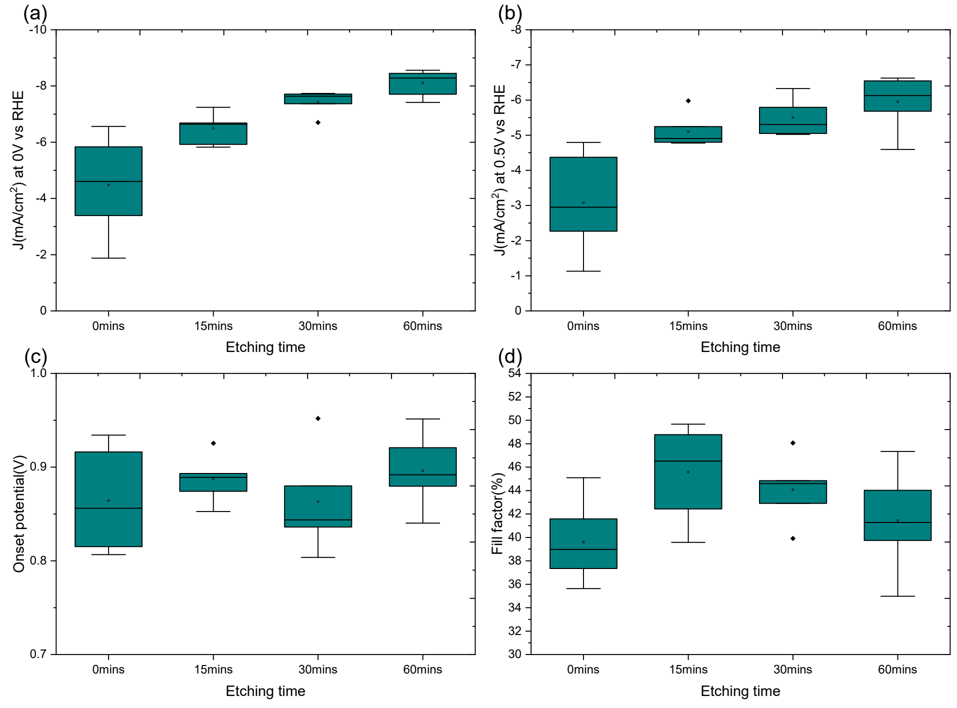

Fig. S 2 The statistical data of the PEC performance obtained in TO-Cu_2O photocathodes with different etching times (0, 15, 30, and 60 minutes). For each group, six samples were counted. Photocurrent density at (a) 0 V_{RHE} and (b) 0.5 V_{RHE}. (c) Onset potential. (d) Fill factor.

Table S 1 Summary of the PEC Performance for Cu_2O-Based Photocathodes

Photocathode architecture	Deposition method	J at 0 V_{RHE} (mA cm^{-2})	J at 0.5 V_{RHE} (mA cm^{-2})	V_{onset} vs RHE (V)	ABPE at V_{RHE}	Ref.
FTO/Au/Cu_2O/AZO/TiO_2/Pt	ED/ALD	5.7*	--	+0.40*	0.66 % at 0.18 V*	Paracchino 2012[6]
FTO/Au/Cu_2O/AZO/TiO_2/RuOx	ED/ALD	5.2*	0.3*	+0.55*	1.05 % at 0.3 V	Azevedo 2014[7]
FTO/Au/Cu_2O/AZO/TiO_2/Pt	ED/ALD	5.5	2.1*	+0.64	1.35 % at 0.45 V*	Azevedo 2014[7]
Au/Cu_2O/ZnS/TiO_2/Pt	TO/TE/ALD	2.3*	1.2*	+0.72	0.6 % at 0.5 V*	Dai 2014[8]
Cu/Cu_2O/Ga_2O_3/TiO_2/Pt	ChO/ALD	2.95	1.5*	+1.02	0.78 % at 0.45 V	Li 2015[9]

FTO/Cu/Cu_2O/AZO/TiO_2/RuOx	ChO/ALD	8.0	--	+0.48	0.88 % at 0.15 V*	Luo 2016[10]
Au/Cu_2O/Ga_2O_3/TiO_2/RuOx	TO/ALD	6.0	3.5	+0.90	1.75 % at 0.5 V	Niu 2018[1]
Cu/Cu_2O/Ga_2O_3/TiO_2/RuOx	ChO/ALD	10.0	6	+1.00	3.0 % at 0.5 V*	Pan 2018[11]
Au/CuSCN/Cu_2O/Ga_2O_3/TiO_2/RuOx	ED/ALD	6.5*	5.5	+1.00	3.18 % at 0.6 V	Pan 2020[12]
Au/Cu_2O/Ga_2O_3/TiO_2/RuOx	TO/ALD	8.6	7	+0.92	3.6 % at 0.56 V	This work

* estimated from figures

Abbreviations: AZO: aluminum-doped zinc oxide; FTO: fluorine-doped tin oxide; ALD: atomic layer deposition; ED: electrodeposition; TO: thermal oxidation; TE: thermal evaporation; ChO: chemical oxidation (then annealing).

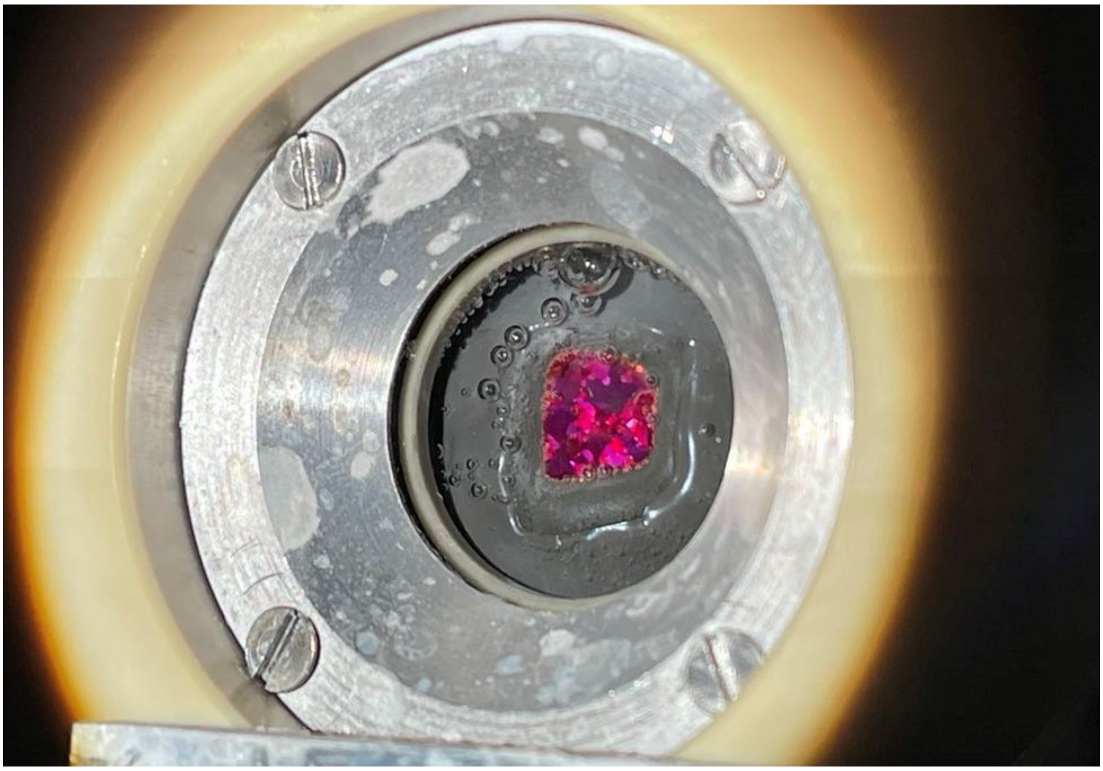

Fig. S 3 Optical image of a TO-Cu_2O photocathode working at 0.5 V_{RHE} under 1 sun.

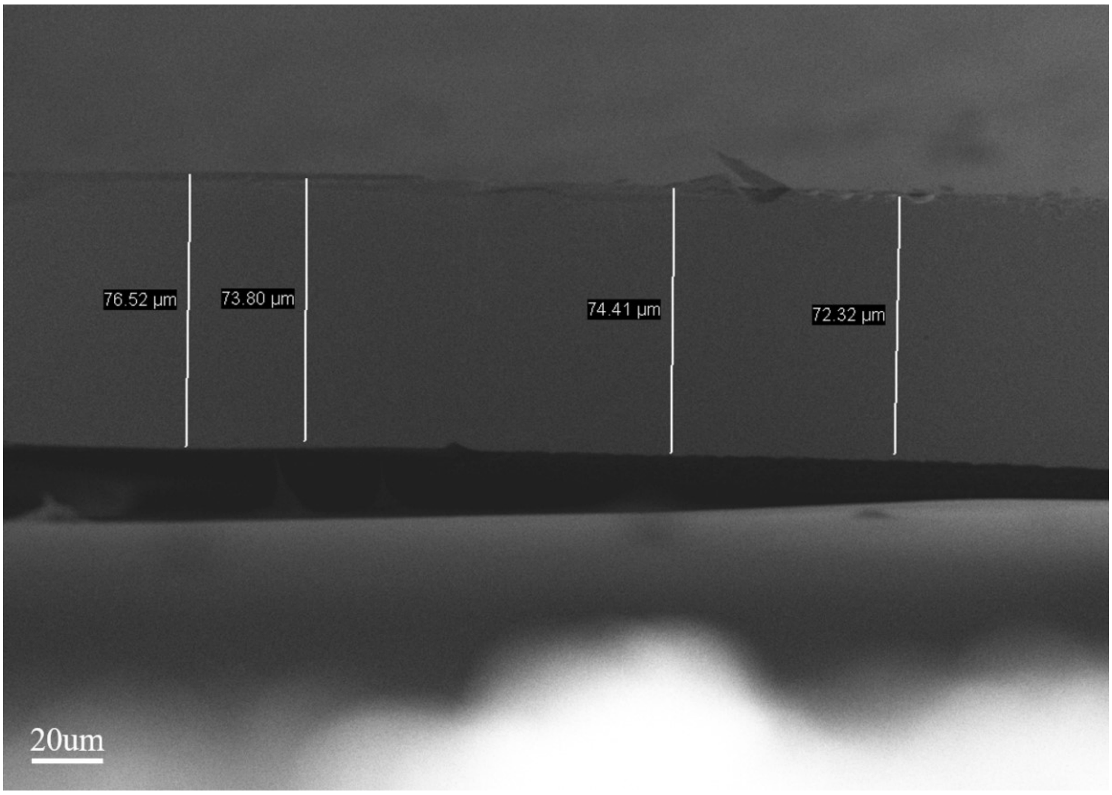

Fig. S 4 The cross-sectional SEM of a TO-Cu_2O sample after etching in ammonia solution for 60 minutes.

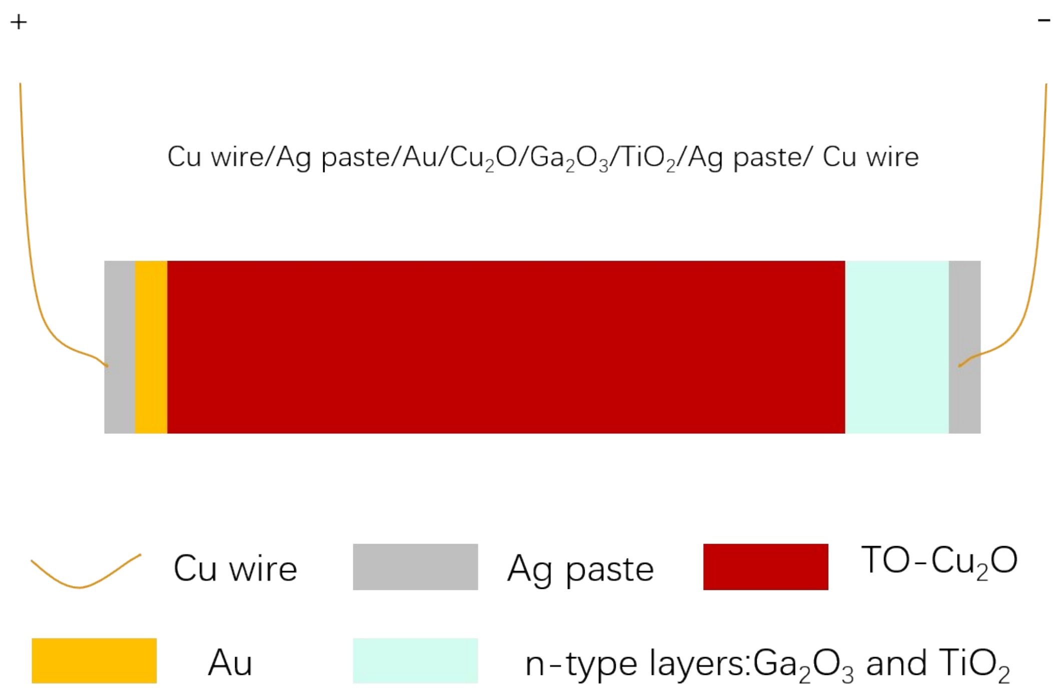

Fig. S 5 The device structure for CV and DLCP measurement.

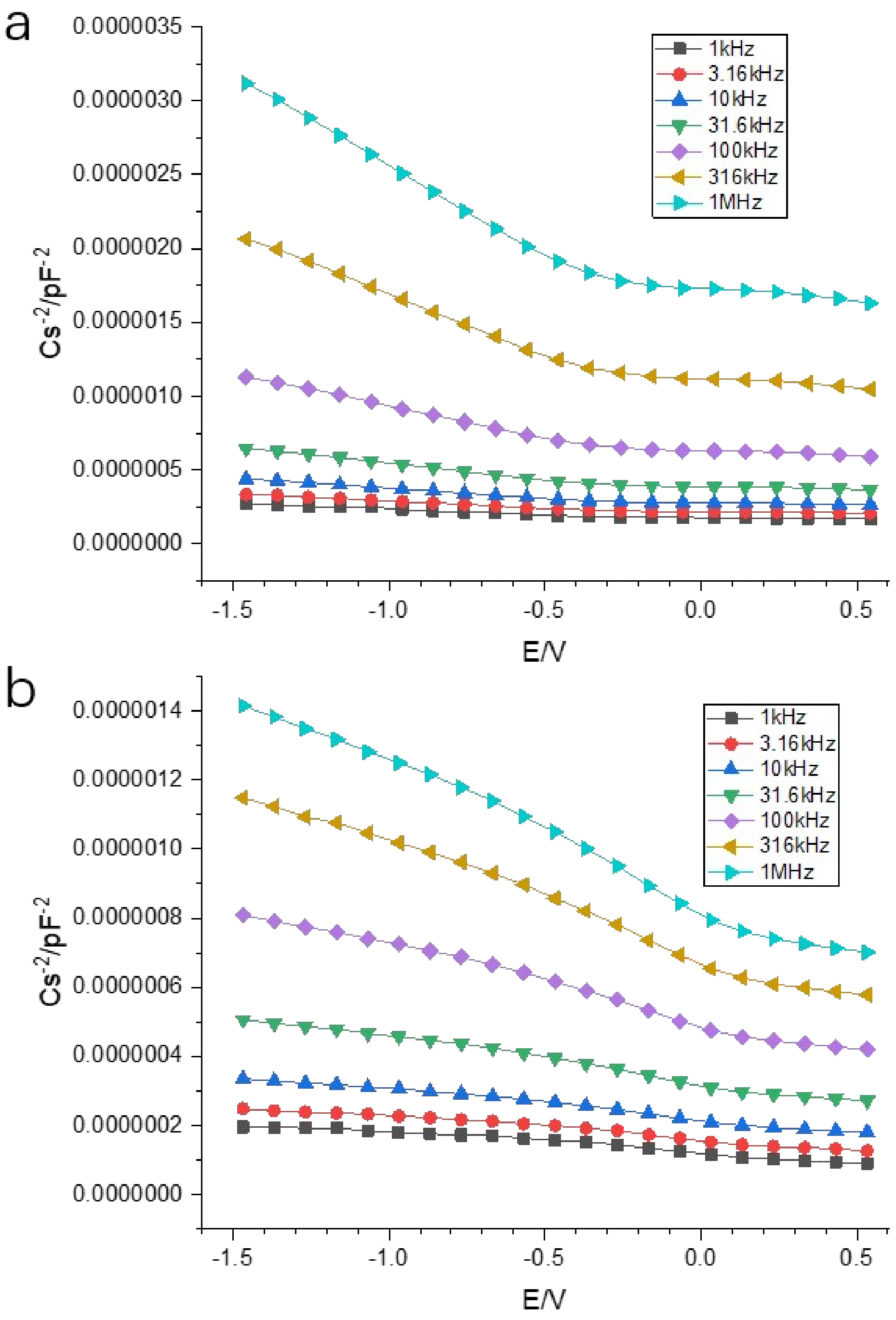

Fig. S 6 The capacitance-voltage scan with the DC bias from -1.5 V to 0.5 V and AC amplitude of 5 mV on (a) unetched and (b) etched sample.

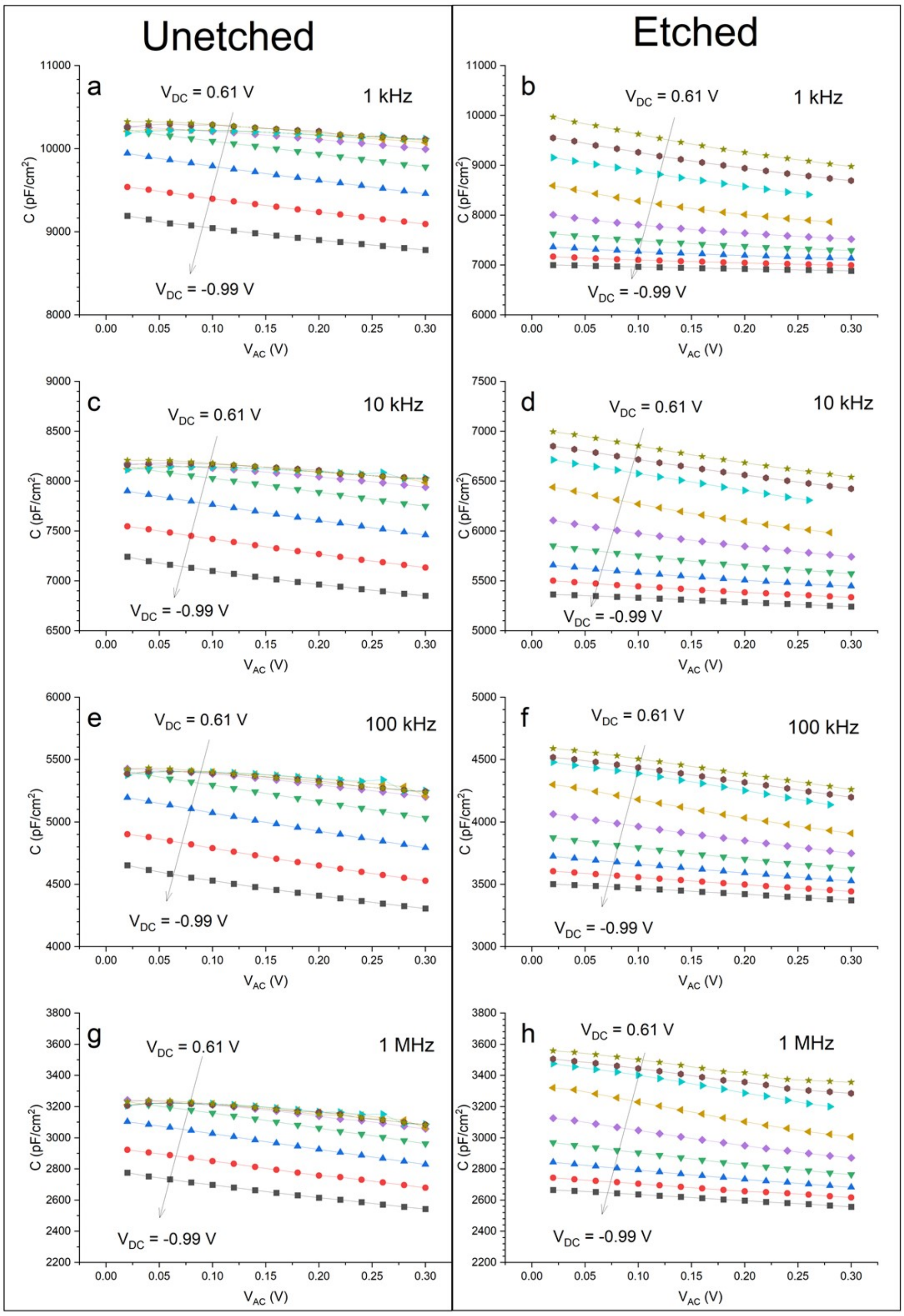

Fig. S 7 Variation of the junction capacitance (C) of unetched (a, c, e, g) and etched (b, d, f, h) devices with respect to the amplitude of the AC biases under different DC biases (from top to bottom, V_{DC} change from 0.61 V to -0.99 V with a step of 0.2 V) measured at AC frequencies of 1 kHz (a, b), 10 kHz (c, d), 100 kHz (e, f), and 1 MHz (g, h). In normal data process, a quadratic function $C = C_0 + C_1 V_{Ac} + C_2 (V_{AC})^2$ was used to fit C and V_{AC} to get C_0 and C_1. (Normally, C_0 is positive, C_1 is negative) However, in the unetched sample, some concave curves will result a positive C_0 and C_1, which will make the calculated N_{DL} negative. In these cases, a linear fitting $C = C_0 + C_1 V_{Ac}$ was used instead of a quadratic fitting to get C_0 and C_1.

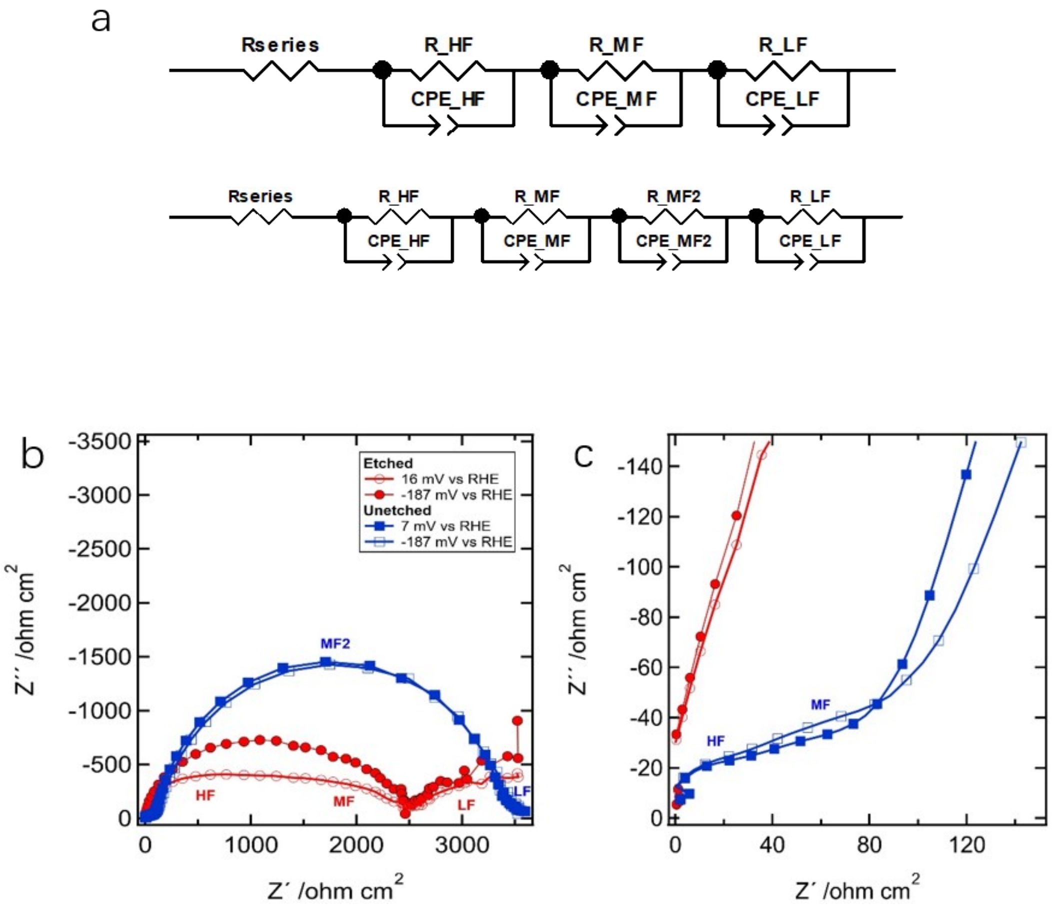

Fig. S 8 (a) The equivalent circuit used for the impedance fitting (upper: etched; lower: unetched). Nyquist plot of the etched and unetched samples at ~0 V vs RHE and ~-200 mV vs RHE. (b) All frequencies, (c) zoom in on the high-frequency region.

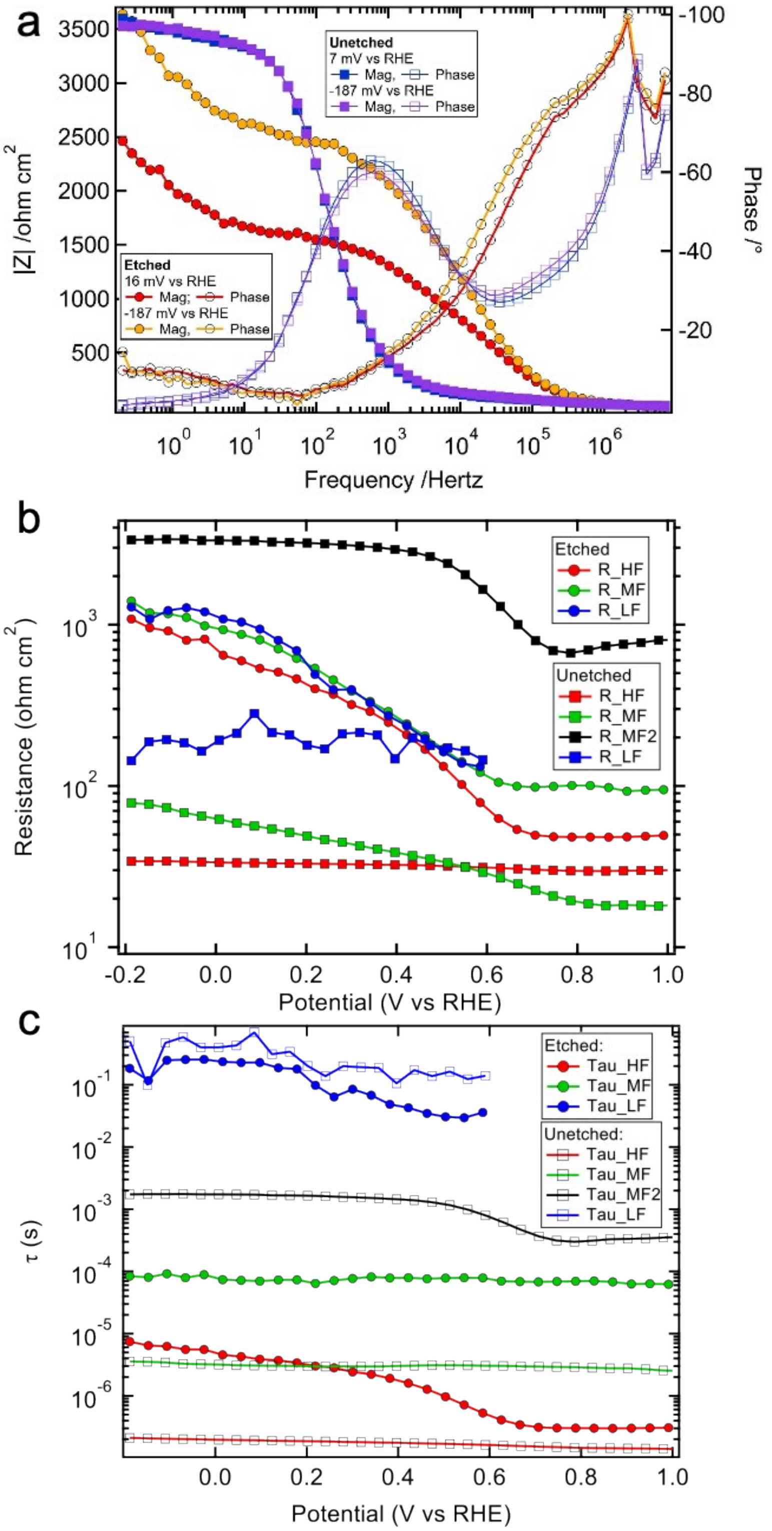

Fig. S 9 (a) Bode plot of the data presented in the Nyquist plot of Fig. S8 (b). (b) Resistances that are present after the photocurrent onset. HF: high frequency, MF: medium frequency, MF2: medium frequency 2 (only present in the unetched sample), LF: low frequency. (c) Time constants calculated by τ=CxR for the different elements from the fitting.

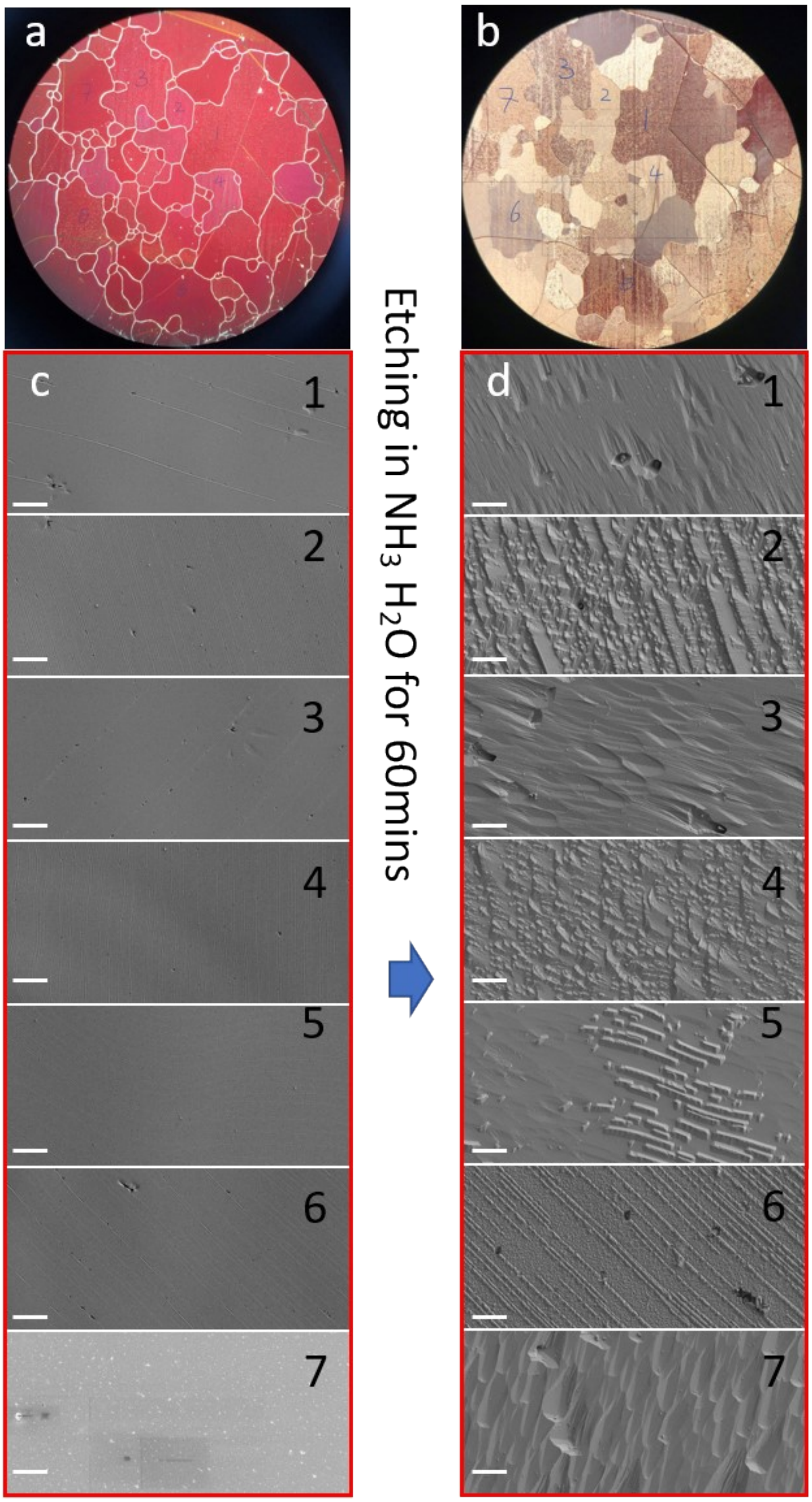

Fig. S 10 Images by light microscope of TO-Cu_2O before (a) and after (b) ammonia solution etching 60 minutes. SEM images of TO-Cu_2O surface at different crystal sites (marked in (a) and (b)) before (c) and after (d) etching for 60 minutes. The scale bar in (c) and (d) is 10 um.

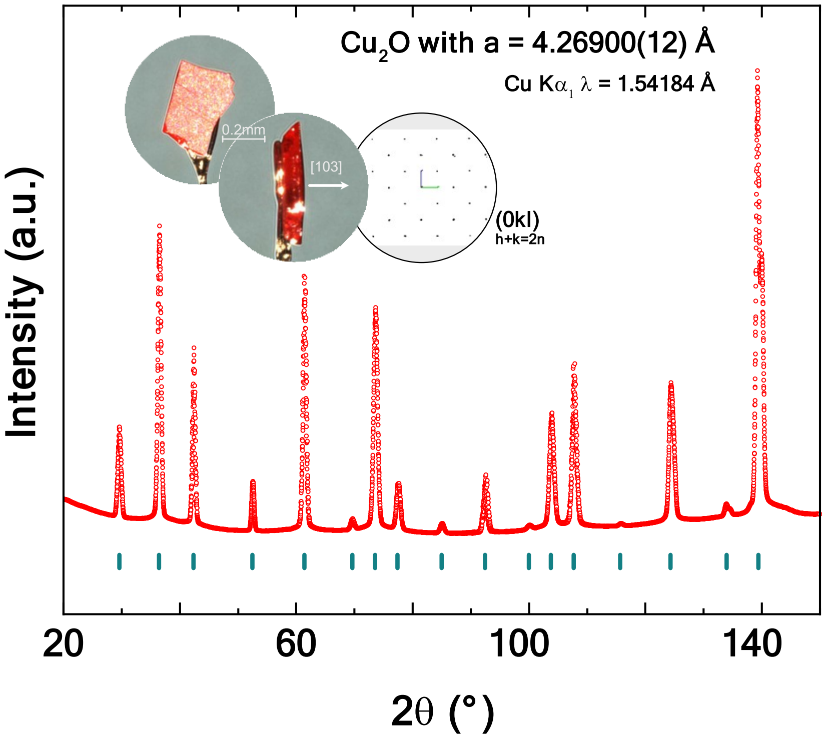

Fig. S 11 2-D powder X-ray diffraction pattern plot extracted from single-crystal X-ray diffraction data using Cu Ka1 radiation with wavelength of 1.54184 Å. Red circles and blue vertical bars correspond to measured diffracted intensities and Bragg position for Cu2O, respectively. Two rotated images of the measured single-crystal are shown and the normal direction is estimated to be close to [103]. The (0kl) plane generated from reconstructing the reciprocal space is shown, which agrees with the Pn-3m space-group.

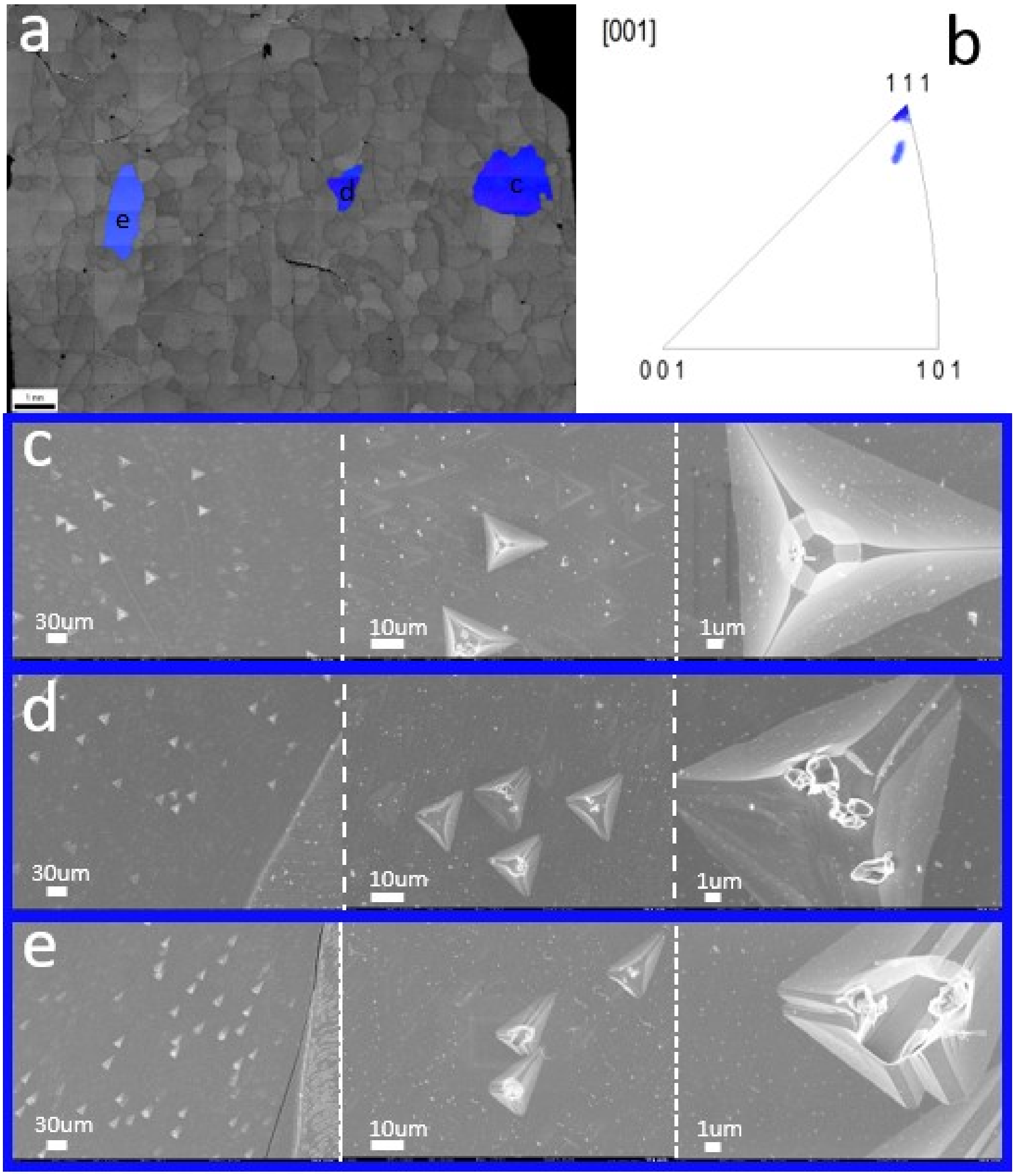

Fig. S 12 (a) The position of three typical crystals with [111] orientation on the sample. (b) The crystal orientation of these three sites shown in inverse pole figure with respect to surface normal direction. (c-d) The morphology of these three crystals under different magnification views.

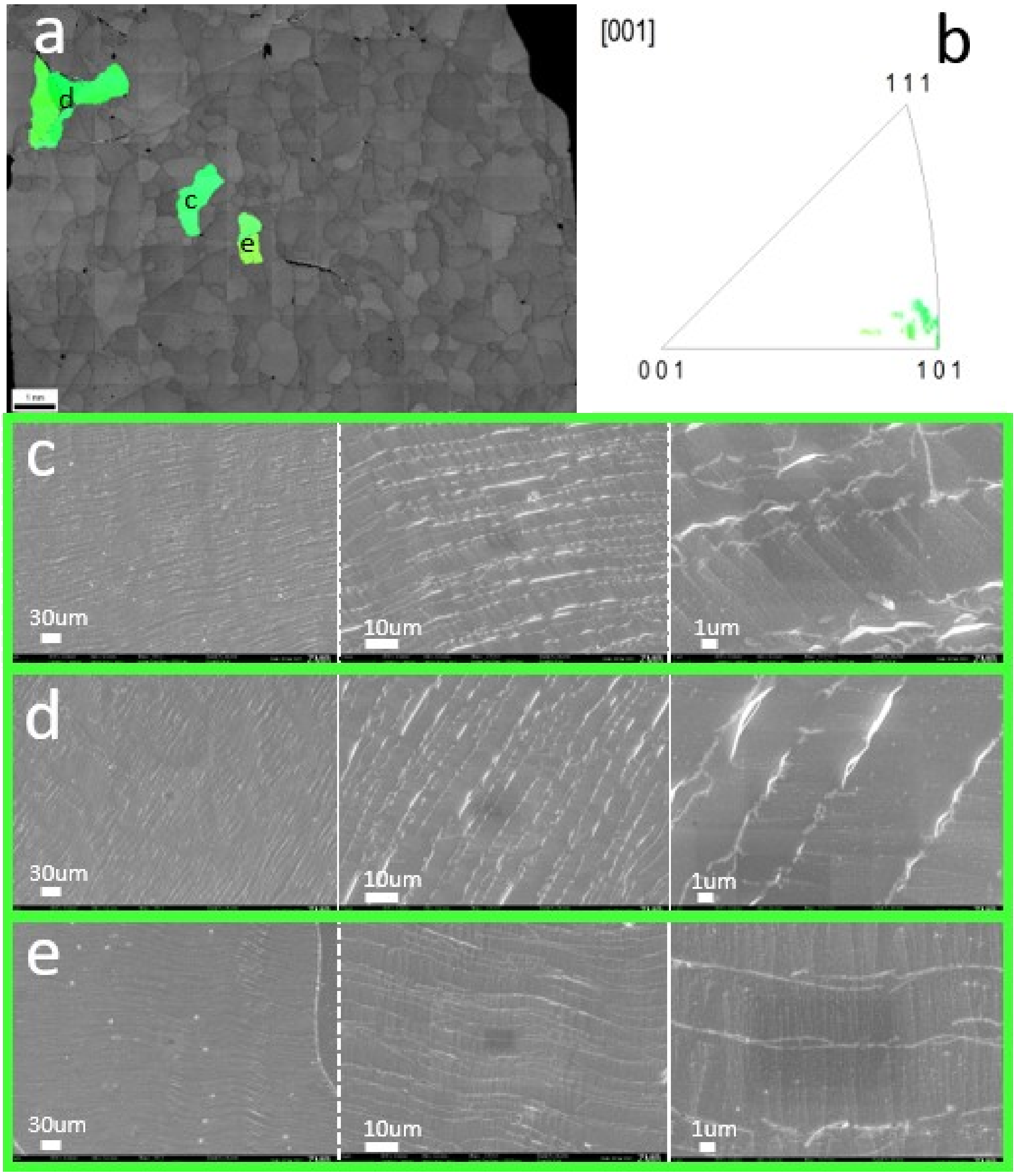

Fig. S 13 (a) The position of three typical crystals with [101] orientation on the sample. (b) The crystal orientation of these three sites shown in inverse pole figure with respect to surface normal direction. (c-d) The morphology of these three crystals under different magnification views.

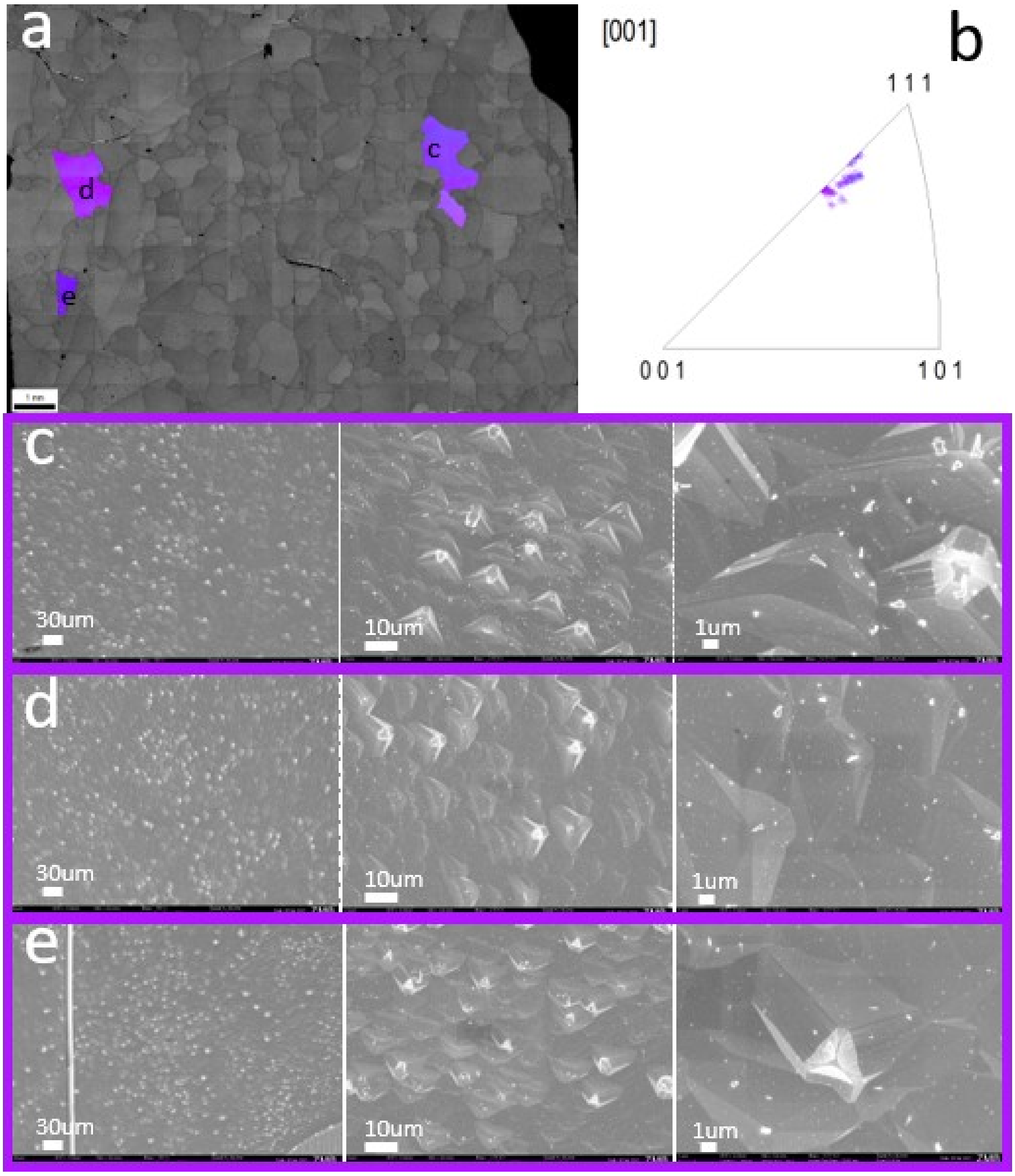

Fig. S 14 (a) The position of three typical crystals with [112]/[223] orientation on the sample. (b) The crystal orientation of these three sites shown in inverse pole figure with respect to surface normal direction. (c-d) The morphology of these three crystals under different magnification views.

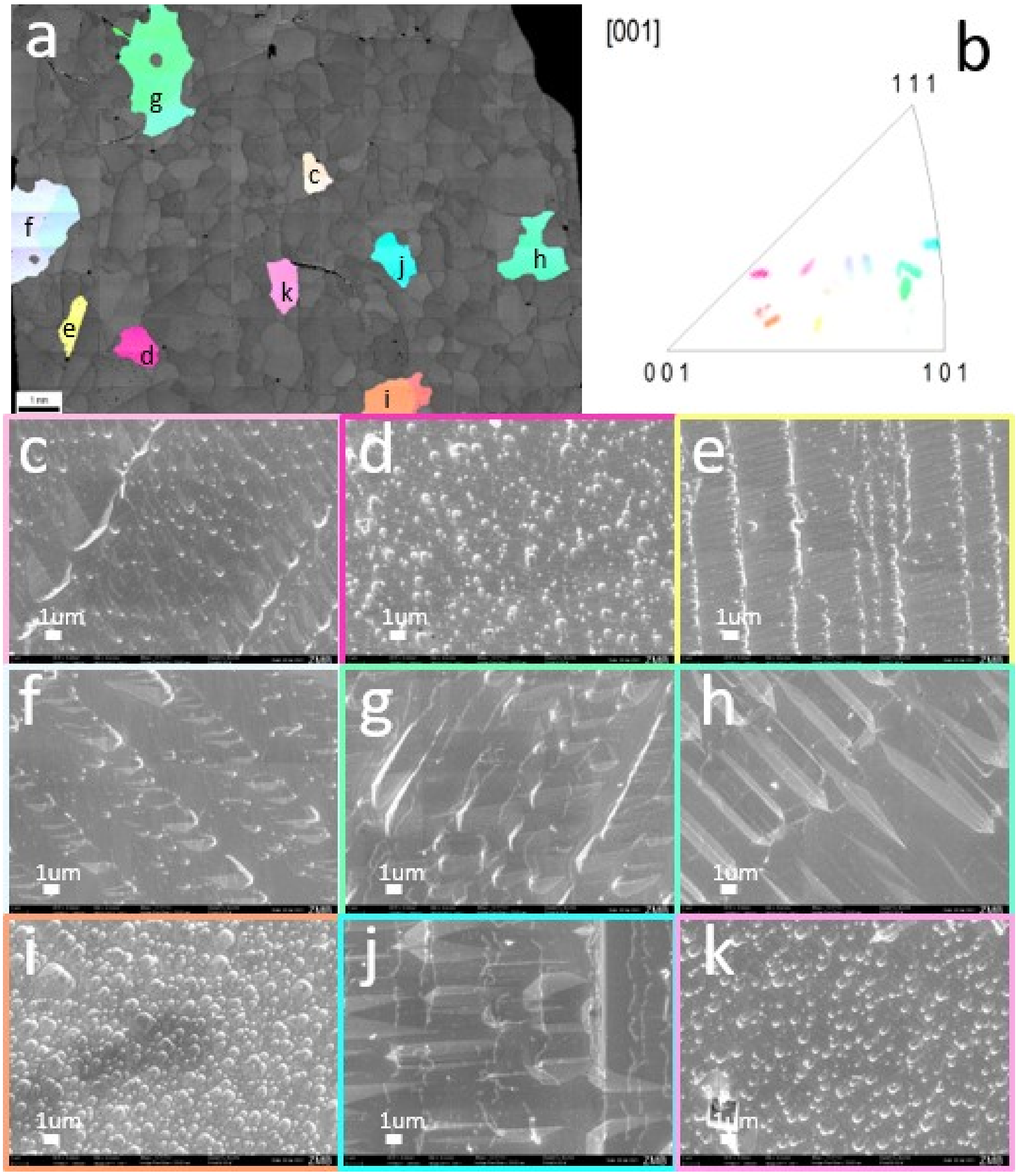

Fig. S 15 (a) The position of nine typical sites with high-index orientation on the sample. (b) The crystal orientation of these nine sites shown in inverse pole figure with respect to surface normal direction. (c-k) The morphology of etch pit for these nine crystals. The orientation indexes of (c) to (k) are [5,2,9], [4,3,13], [5,1,10], [4,2,7], [4,1,5], [6,2,7], [4,1,12], [5,2,5], and [4,2,9] respectively.

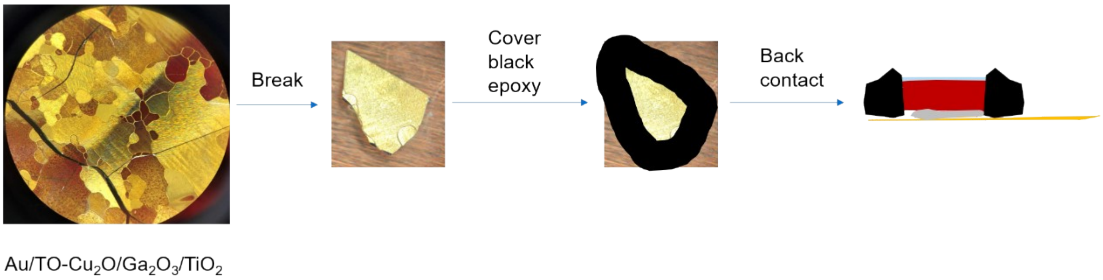

Fig. S 16 Fabrication procedure of single-crystal TO-Cu_2O photocathodes.

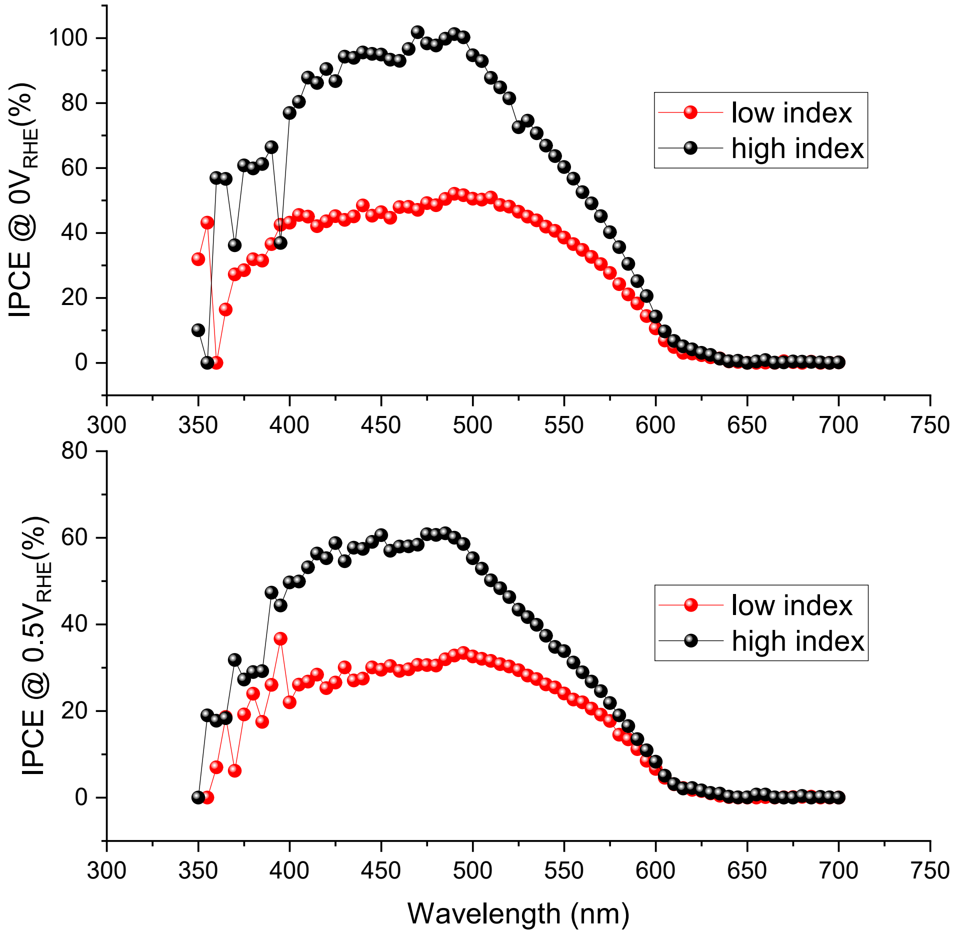

Fig. S 17 The IPCE of single-crystal photocathodes at 0 V_{RHE} and 0.5 V_{RHE}.

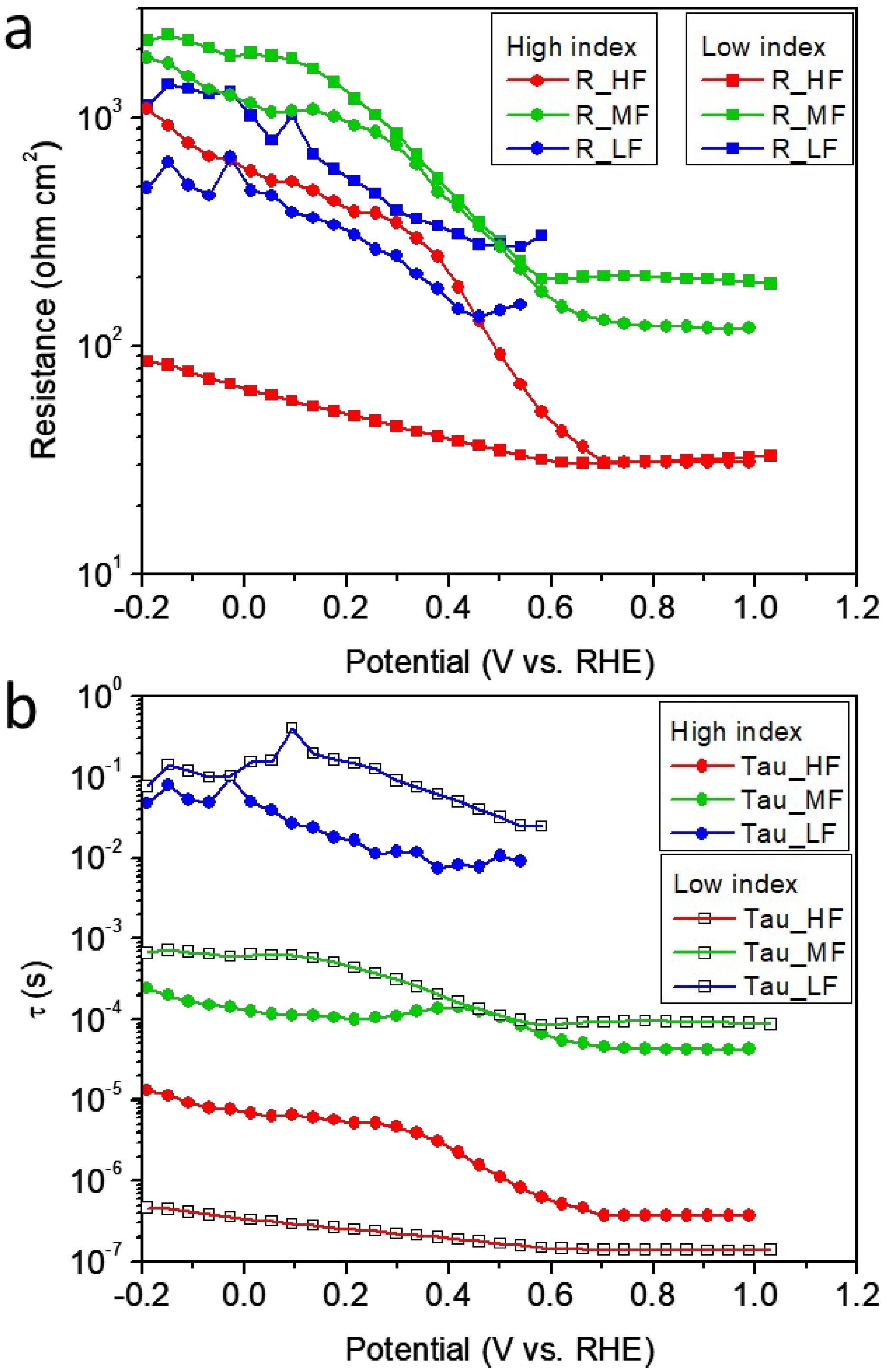

Fig. S 18 (a) The extracted resistances from the equivalent circuit in Fig. S8. (b) The time constants calculated by the extracted capacitances and resistances.

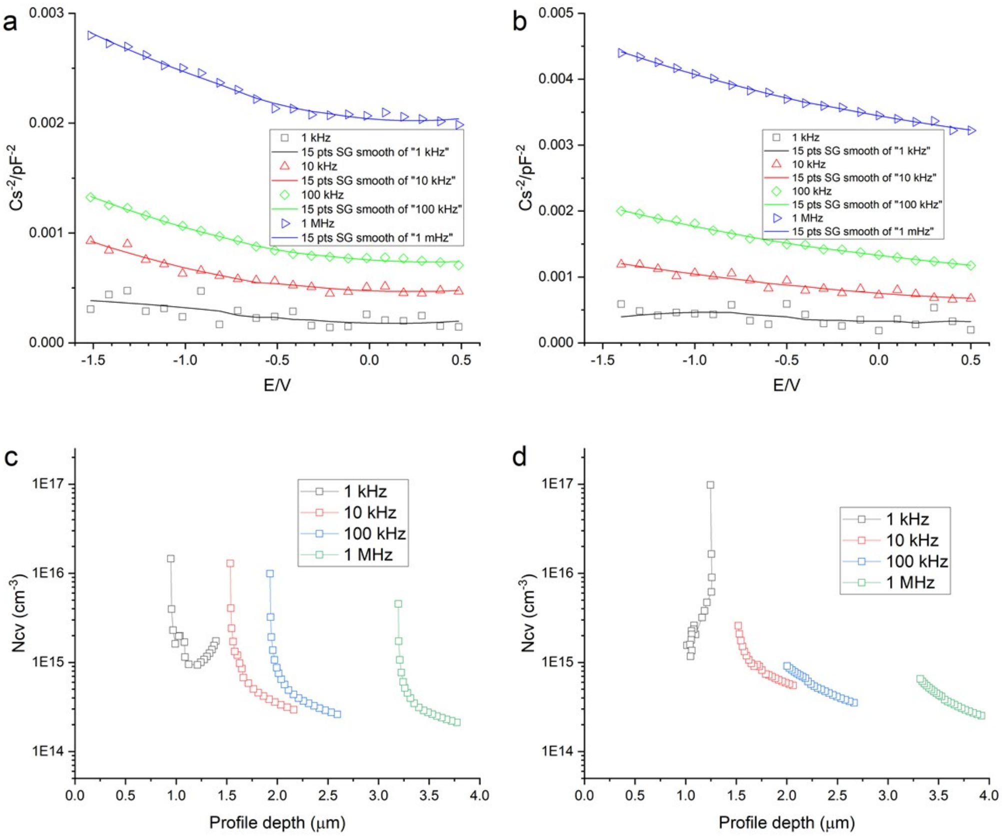

Fig. S 19 The capacitance-voltage scan with a DC bias from -1.5 V to 0.5 V and an AC amplitude of 5 mV on the low-index (a) and high-index (b) samples. The derived N_{CV}-profile depth <x> plots from a C-V scan of low index (c) and high index (d) samples. Since the capacitance measured in the single crystal devices are noisy, especially at low frequency, Savitzky-Golay smoothing was used to calculate the $d(Cs^{-2})/d(V_{DC})$, and the profiling depth was calculated based on the smoothed capacitance.

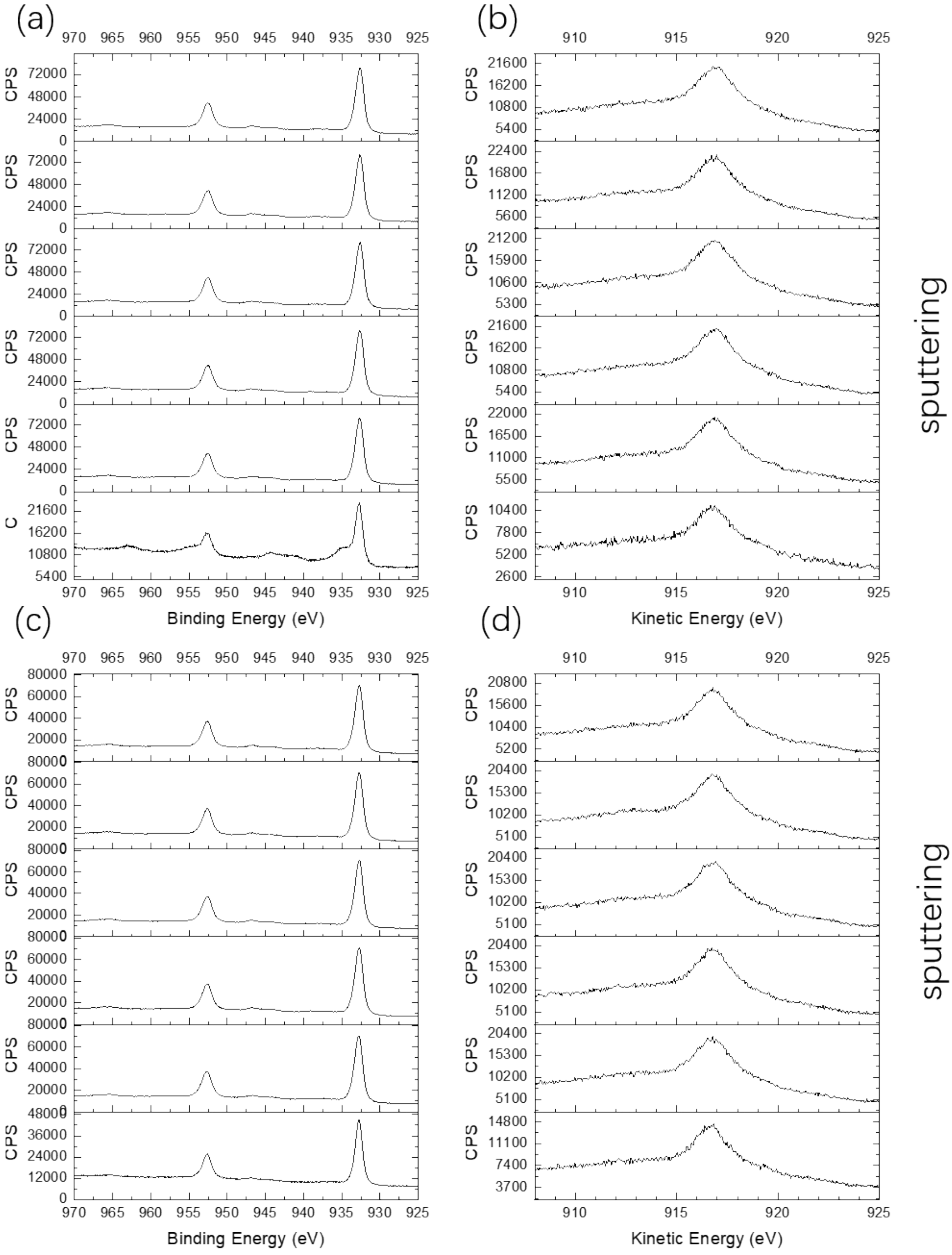

Fig. S 20 The (a)(c) Cu 2p and (b)(d) Cu LMM Auger peaks in depth profile XPS measurement with the (a)(b) as-grown and (c)(d) etched bare TO-Cu_2O samples.

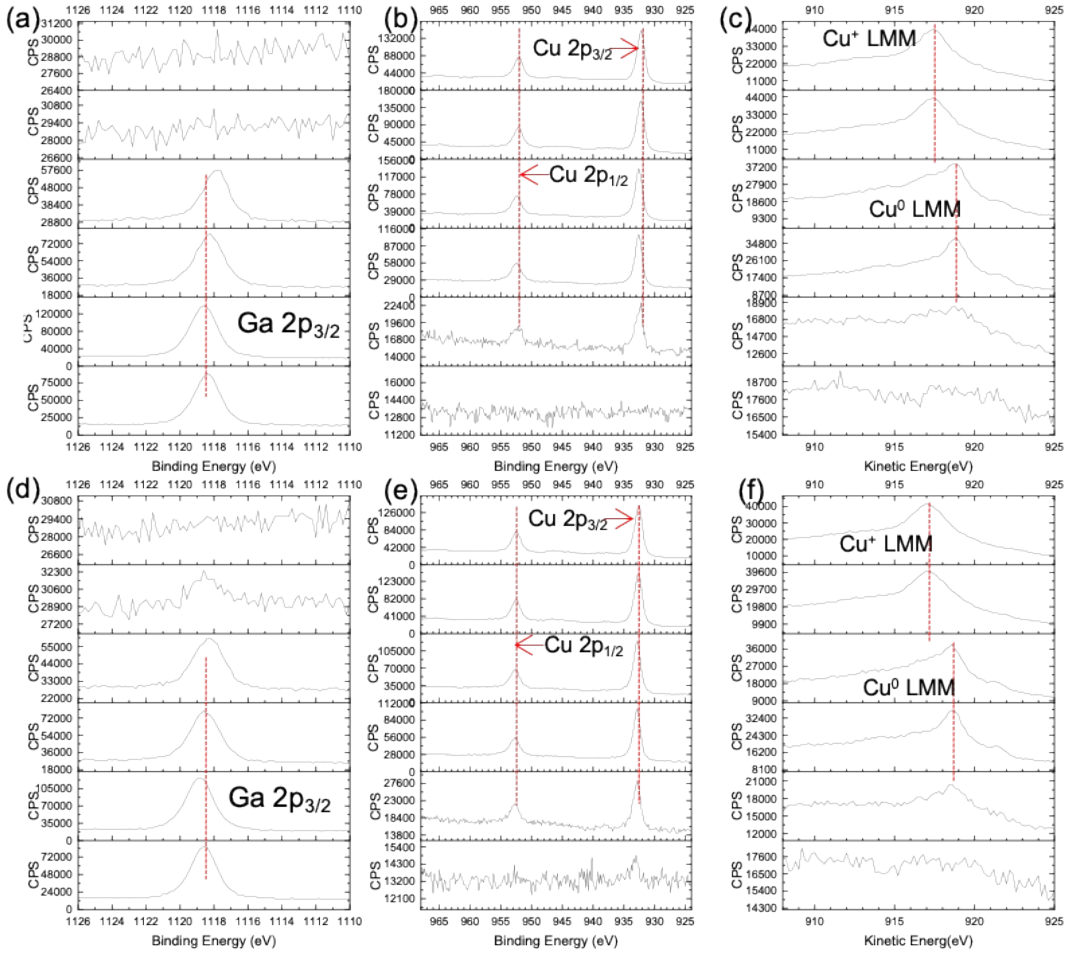

Fig. S 21 The (a)(d) Ga 2p, (b)(e) Cu 2p, and (c)(f) Cu LMM Auger peaks in depth profile XPS measurement with the (a)(b)(c) as-grown and (d)(e)(f) etched TO-Cu_2O/Ga_2O_3(5nm) samples.

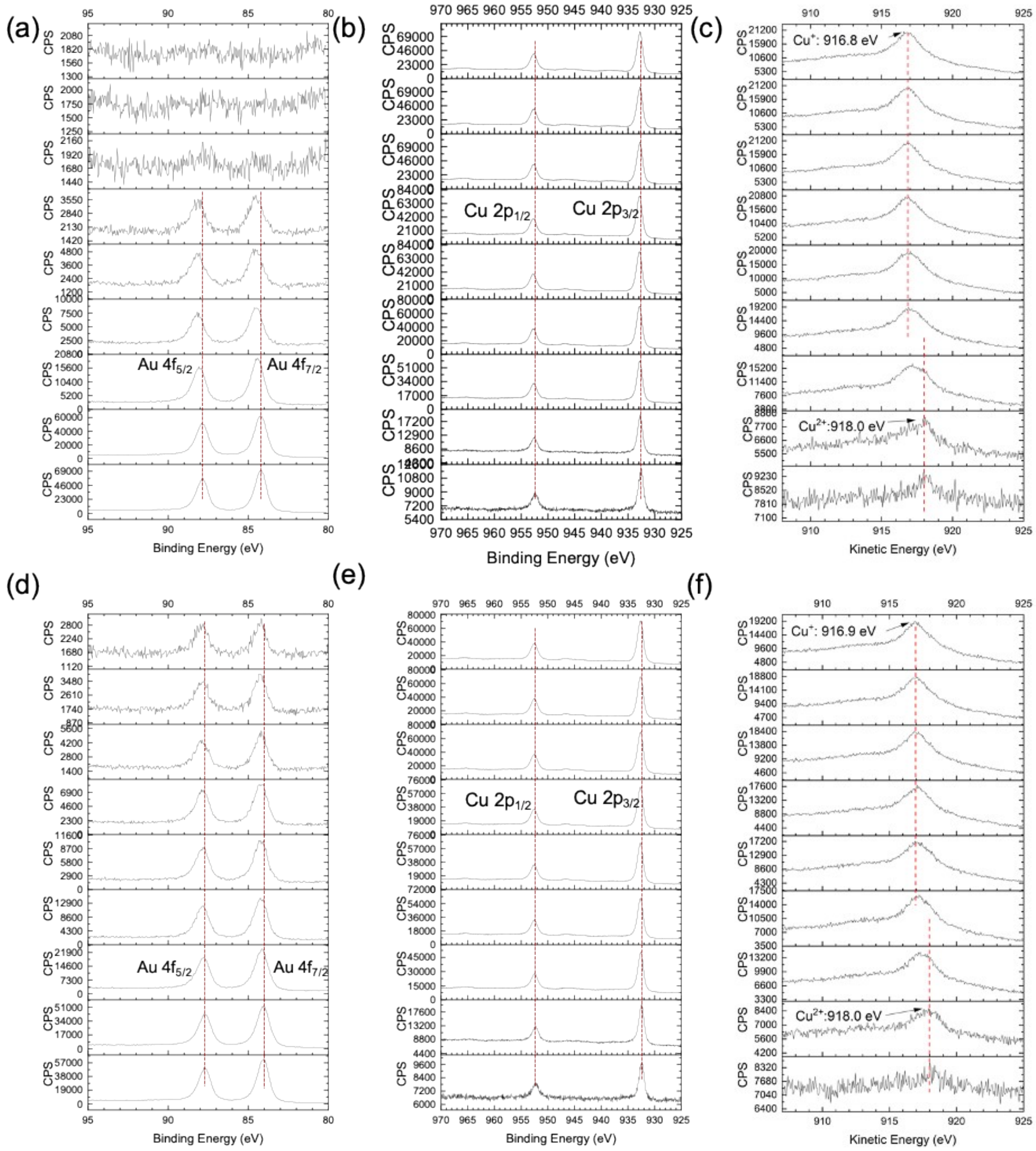

Fig. S 22 The (a)(d) Au 4f, (b)(e) Cu 2p, and (c)(f) Cu LMM Auger peaks in depth profile XPS measurement with the (a)(b)(c) as-grown and (d)(e)(f) etched TO-Cu_2O/Au(5nm) samples.

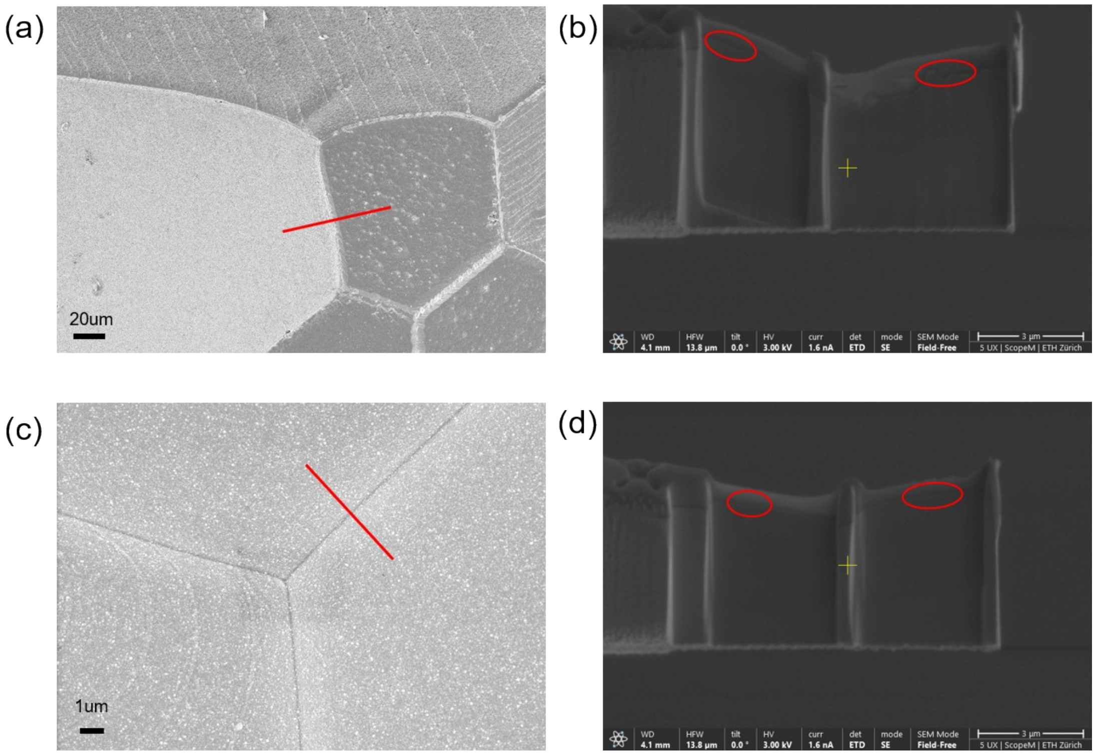

Fig. S 23 The cutting position by FIB on (a) etched and (c) unetched sample. The red lines show where the TEM lamellae come from. The SEM images of TEM lamellae of (b) etched and (d) unetched devices. The red circle showed where the STEM-EDS was performed.

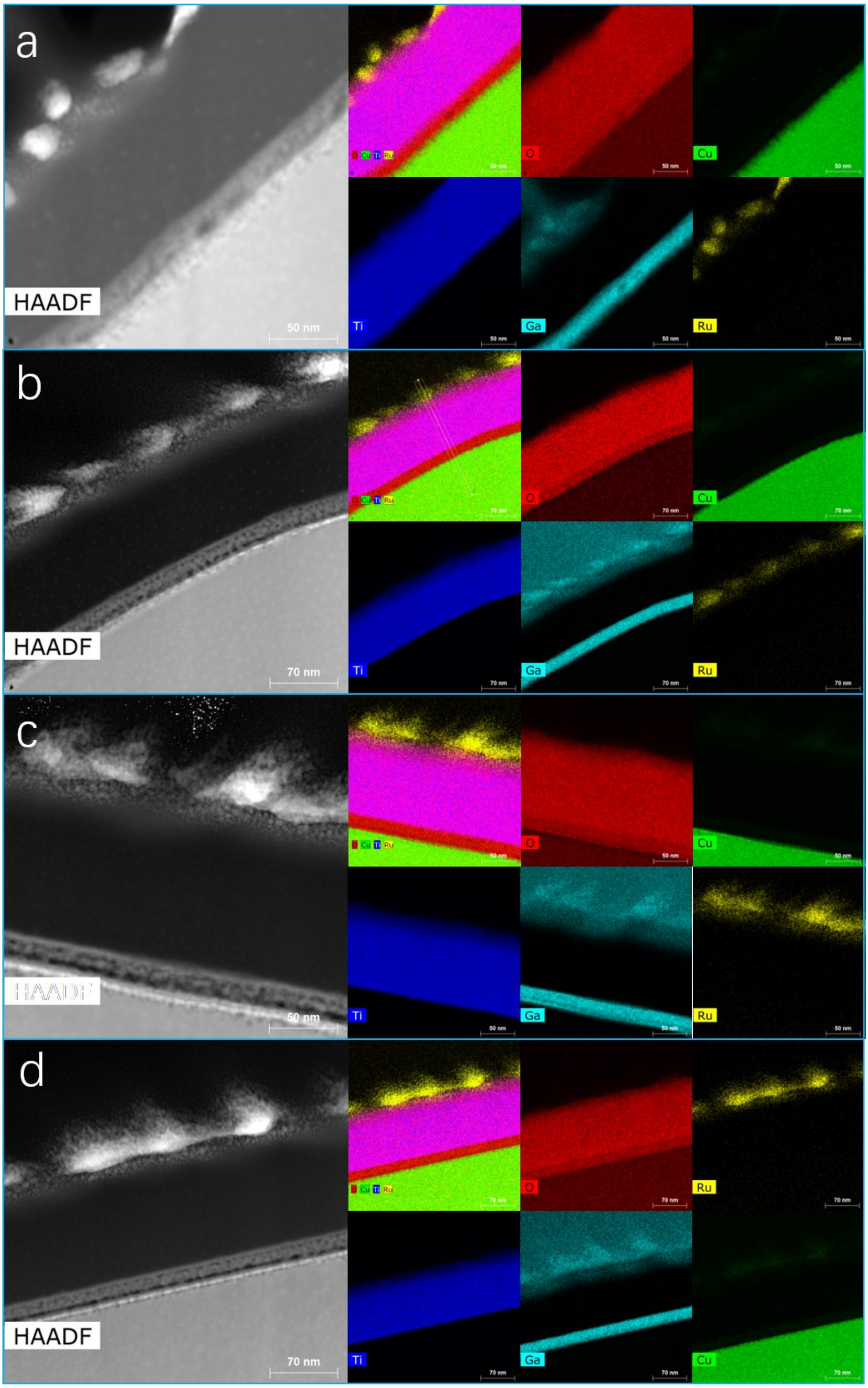

Fig. S 24 The HAADF image and EDS mapping of high index area (a) and low index area (b) of the etched device, site1 (c) and site2 (d) of the unetched device.

Table S 2 The summary of the copper chemical state at the surface of Cu_2O.

**Sample type**	**Etched**		**Unetched**	
**Site**	High index area	Low index area	Site1	Site2
Metallic copper observed by XPS at the surface of bare Cu_2O	NO		NO	
CuO observed by XPS at the surface of bare Cu_2O	No		Yes	
metallic copper observed by XPS at the interface of Cu_2O/Ga_2O_3(5nm)	Yes		Yes	
CuO observed by XPS at the interface of Cu_2O/Ga_2O_3(5nm)	No		No	
metallic copper observed by XPS at the interface of Cu_2O/Au(5nm)	No		No	
metallic copper observed in STEM (or HAADF and EDS mapping)	No	Yes	Yes	Yes

Supplementary references

1. W. Niu, T. Moehl, W. Cui, R. Wick-Joliat, L. Zhu and S. D. Tilley, *Advanced Energy Materials*, 2018, **8**, 1702323.
2. S. D. Tilley, M. Schreier, J. Azevedo, M. Stefik and M. Graetzel, *Advanced Functional Materials*, 2014, **24**, 303-311.
3. D. Huang, K. Wang, L. Li, K. Feng, N. An, S. Ikeda, Y. Kuang, Y. Ng and F. Jiang, *Energy & Environmental Science*, 2021, **14**, 1480-1489.
4. E. C. Heltemes, *Physical Review*, 1966, **141**, 803-805.
5. Z. Ni, C. Bao, Y. Liu, Q. Jiang, W.-Q. Wu, S. Chen, X. Dai, B. Chen, B. Hartweg, Z. Yu, Z. Holman and J. Huang, *Science*, 2020, **367**, 1352-1358.
6. A. Paracchino, N. Mathews, T. Hisatomi, M. Stefik, S. D. Tilley and M. Grätzel, *Energy & Environmental Science*, 2012, **5**, 8673-8681.
7. J. Azevedo, L. Steier, P. Dias, M. Stefik, C. T. Sousa, J. P. Araújo, A. Mendes, M. Graetzel and S. D. Tilley, *Energy & Environmental Science*, 2014, **7**, 4044-4052.
8. P. Dai, W. Li, J. Xie, Y. He, J. Thorne, G. McMahon, J. Zhan and D. Wang, *Angewandte Chemie International Edition*, 2014, **53**, 13493-13497.
9. C. Li, T. Hisatomi, O. Watanabe, M. Nakabayashi, N. Shibata, K. Domen and J.-J. Delaunay, *Energy & Environmental Science*, 2015, **8**, 1493-1500.
10. J. Luo, L. Steier, M.-K. Son, M. Schreier, M. T. Mayer and M. Grätzel, *Nano Letters*, 2016, **16**, 1848-1857.
11. L. Pan, J. H. Kim, M. T. Mayer, M.-K. Son, A. Ummadisingu, J. S. Lee, A. Hagfeldt, J. Luo and M. Grätzel, *Nature Catalysis*, 2018, **1**, 412-420.
12. L. Pan, Y. Liu, L. Yao, R. Dan, K. Sivula, M. Grätzel and A. Hagfeldt, *Nature Communications*, 2020, **11**, 318.
